# RNA-seq and Network Analysis Reveal Unique Chemokine Activity Signatures in the Synovial Tissue of Patients With Rheumatoid Arthritis

**DOI:** 10.3389/fmed.2022.799440

**Published:** 2022-05-04

**Authors:** Runrun Zhang, Yehua Jin, Cen Chang, Lingxia Xu, Yanqin Bian, Yu Shen, Yang Sun, Songtao Sun, Steven J. Schrodi, Shicheng Guo, Dongyi He

**Affiliations:** ^1^Department of Rheumatology, Shanghai Guanghua Hospital, Shanghai University of Traditional Chinese Medicine, Shanghai, China; ^2^Department of Rheumatology, The Second Affiliated Hospital of Shandong University of Traditional Chinese Medicine, Jinan, China; ^3^Guanghua Clinical Medical College, Shanghai University of Traditional Chinese Medicine, Shanghai, China; ^4^Guanghua Integrative Medicine Hospital, Shanghai, China; ^5^Arthritis Institute of Integrated Traditional and Western Medicine, Shanghai Chinese Medicine Research Institute, Shanghai, China; ^6^Department of Orthopedics, Shanghai Guanghua Hospital, Shanghai University of Traditional Chinese Medicine, Shanghai, China; ^7^Department of Medical Genetics, School of Medicine and Public Health, University of Wisconsin-Madison, Madison, WI, United States

**Keywords:** rheumatoid arthritis, osteoarthritis, synovial tissue, RNA-seq, differential gene expression

## Abstract

**Purpose:**

This study aimed to provide a comprehensive understanding of the genome-wide expression patterns in the synovial tissue samples of patients with rheumatoid arthritis (RA) to investigate the potential mechanisms regulating RA occurrence and development.

**Methods:**

Transcription profiles of the synovial tissue samples from nine patients with RA and 15 patients with osteoarthritis (OA) (control) from the East Asian population were generated using RNA sequencing (RNA-seq). Gene set enrichment analysis (GSEA) was used to analyze all the detected genes and the differentially expressed genes (DEGs) were identified using DESeq. To further analyze the DEGs, the Gene Ontology (GO) functional enrichment and the Kyoto Encyclopedia of Genes and Genomes (KEGG) pathway analyses were performed. The protein–protein interaction (PPI) network of the DEGs was constructed using the Search Tool for the Retrieval of Interacting Genes/Proteins (STRING) and the hub genes were identified by topology clustering with the Molecular Complex Detection (MCODE)-Cytoscape. The most important hub genes were validated using quantitative real-time PCR (qRT-PCR).

**Results:**

Of the 17,736 genes detected, 851 genes were identified as the DEGs (474 upregulated and 377 downregulated genes) using the false discovery rate (FDR) approach. GSEA revealed that the significantly enriched gene sets that positively correlated with RA were CD40 signaling overactivation, Th1 cytotoxic module, overactivation of the immune response, adaptive immune response, effective vs. memory CD8+ T cells (upregulated), and naïve vs. effective CD8+ T cells (downregulated). Biological process enrichment analysis showed that the DEGs were significantly enriched for signal transduction (*P* = 3.01 × 10^−6^), immune response (*P* = 1.65 × 10^−24^), and inflammatory response (*P* = 5.76 × 10^−10^). Molecule function enrichment analysis revealed that the DEGs were enriched in calcium ion binding (*P* = 1.26 × 10^−5^), receptor binding (*P* = 1.26 × 10^−5^), and cytokine activity (*P* = 2.01 × 10^−3^). Cellular component enrichment analysis revealed that the DEGs were significantly enriched in the plasma membrane (*P* = 1.91 × 10^−31^), an integral component of the membrane (*P* = 7.39 × 10^−13^), and extracellular region (*P* = 7.63 × 10^−11^). The KEGG pathway analysis showed that the DEGs were enriched in the cytokine–cytokine receptor interaction (*P* = 3.05 × 10^−17^), chemokine signaling (*P* = 3.50 × 10^−7^), T-cell receptor signaling (*P* = 5.17 × 10^−4^), and RA (*P* = 5.17 × 10^−4^) pathways. We confirmed that RA was correlated with the upregulation of the PPI network hub genes, such as *CXCL13, CXCL6, CCR5, CXCR5, CCR2, CXCL3*, and *CXCL10*, and the downregulation of the PPI network hub gene such as *SSTR1*.

**Conclusion:**

This study identified and validated the DEGs in the synovial tissue samples of patients with RA, which highlighted the activity of a subset of chemokine genes, thereby providing novel insights into the molecular mechanisms of RA pathogenesis and identifying potential diagnostic and therapeutic targets for RA.

## Introduction

Rheumatoid arthritis (RA) is an autoimmune disease characterized by synovial inflammation, hyperplasia, and cartilage and bone destruction. Clinical manifestations of RA include joint pain, swelling, stiffness, and deformation (1, 2). RA pathogenesis is thought to be related to genetic and environmental factors, obesity, diet, and gut microbiota composition (3, 4). Osteoarthritis (OA) is a joint disease characterized by the degeneration of the synovial joint and loss of articular cartilage, with primary clinical features including pain and loss of mobility (5). Genetic factors, diet, estrogen level, obesity, bone density, and joint laxity play a role in the pathogenesis of OA (6). As both the RA and OA share common physiological targets, biomarkers present in the synovial tissue that could discriminate between these diseases should be determined (7, 8).

Transcriptomics is tissue specific and it offers an avenue for the investigation of the effects of the disease at the cellular level that is likely to play an important role in the etiology of the disease (9). RNA sequencing (RNA-seq) technology has become the primary step in transcriptomic studies for the characterization of gene expression within cells and tissues. Therefore, identification of differential gene expression in the synovial tissues of patients with RA and OA using RNA-seq may provide new insights into the molecular pathophysiology of these diseases.

In this study, to better understand the functional differences at the transcriptome level between RA and OA, we analyzed the whole genes detected by gene set enrichment analysis (GSEA), identified the differentially expressed genes (DEGs) in the synovial tissue samples of patients with RA and OA using RNA-seq, analyzed the DEGs by the Gene Ontology (GO) functional enrichment and the Kyoto Encyclopedia of Genes and Genomes (KEGG) pathway analyses, constructed the protein–protein interaction (PPI) network, and screened and verified the hub genes. The validated hub genes may serve as critical molecular markers for identifying differences in the synovial tissues of patients with RA and OA due to their central role in gene expression networks.

## Materials and Methods

### Patient Information and Tissue Collection

In this study, we included nine patients with RA, who were diagnosed based on the 2010 American College of Rheumatology (ACR)/European League Against Rheumatism (EULAR) classification criteria for RA (10) and 15 patients with OA, who were diagnosed according to the ACR OA classification criteria (11). The synovial tissue samples of patients with RA and OA were obtained from the Guanghua Hospital, Shanghai, China. All of the involved patients underwent a knee replacement. After removing excess fat and vascular tissue, the synovial tissue samples were placed in liquid nitrogen till further use. The demographic information of patients with RA is given in Table 1 and clinical information of patients with RA and OA is given in Supplementary Table 1. This study was approved by the Ethics Committee of Guanghua Hospital of Integrated Traditional Chinese and Western Medicine (approval number: 2018-K-12) and a written consent was obtained from all the patients prior to knee replacement.

**Table 1:** Demographic information of patients with rheumatoid arthritis (RA).

	**Course** **(month)**	**Age** **(year)**	**Gender**	**H/W** **(cm)/(kg)**	**ESR** **(mm/h)**	**CRP** **(mg/L)**	**RF-IGM** **(IU/ml)**	**RF-IGG** **(U/ml)**	**RF-IGA** **(U/ml)**	**Anti-CCP** **(RU/ml)**
RA1	20	73	F	150/47	65	19.6	25.3	22.39	23.32	326.2
RA2	10	62	F	155/55	40	5.15	9.69	2.2	0.63	<20
RA3	14	61	F	163/62	16	6.28	<20	6.91	188.63	1,600
RA4	20	57	F	150/35	80	178.3	528	198	300	1,197.4
RA5	3	72	M	165/50	53	10.96	45.2	24.15	22.02	1,555.9
RA6	40	70	F	150/45	27	20.68	\	\	\	\
RA7	54	70	F	160/71	48	<0.5	\	\	\	\
RA8	2	64	F	16,360	65	44.28	<10.10	0.15	0.26	20
RA9	7	75	F	160/55	66.76	72.52	\	\	\	\

*H/W, Height/Weight; F, Female; M, Male; ESR, Erythrocyte Sedimentation Rate; CRP, C-reactive protein; RF, Rheumatoid Factor; CCP, Cyclic Citrullinated Peptide Antibody*.

*Smoking: None of the patients, Hypertension: RA7 (10 years)/RA8 (20 years)/RA9 (5 years), Diabetes: RA7 (3 years)*.

### Ribonucleic Acid Isolation and Library Preparation

Total RNA was extracted from the synovial tissue samples using TRIzol Reagent (Thermo Fisher Scientific, Waltham, Massachusetts, USA) according to the manufacturer's protocol. A NanoDrop 2000 Spectrophotometer (Thermo Fisher Scientific) was used to evaluate RNA quality and quantify each RNA sample. RNA integrity was assessed using the Agilent 2100 Bioanalyzer (Agilent Technologies, Santa Clara, California, USA). Total RNA with a standard RNA integrity number (RIN) ≥ 7.0 and 28S/18S ≥ 0.7 was subjected to RNA-seq. RNA libraries were constructed using the TruSeq Stranded mRNA LT Sample Prep Kit (Illumina, San Diego, California, USA) according to the manufacturer's instructions.

### Ribonucleic Acid Sequencing and Identification of the Differentially Expressed Genes

The libraries were sequenced on an Illumina HiSeq × 10 platform. Raw data (raw reads) in FASTQ format were first processed using Trimmomatic (12) and low-quality reads were removed to obtain clean reads. Clean reads were then mapped to the human genome (GRCh38) using HISAT2 (13). The fragments per kilobase of transcript per millions mapped reads (FPKM) of each gene were calculated using Cufflinks (14, 15) and the read counts of each gene were obtained using HTSeq-Count (16). Differential expression analysis was performed using the DESeq R package (17). An adjusted *P* < 0.05 and |log2FoldChange| ≥ 1.5 were set as the threshold for significant differential expression; *P*-value was adjusted using the false discovery rate (FDR). The expression profiling data were obtained from https://github.com/dongyihe/rheumatoidarthritis.

### Gene Set Enrichment Analysis Based on RNA Sequencing Effectively Detected Genes

Gene set enrichment analysis was performed using a defined set of genes to determine statistically significant differences between the RA and OA groups, using R software (https://www.r-project.org) and the data set was obtained from the Molecular Signatures Database version 7.2 (MSigDB; GSEA-MSigDB website). MSigDB is a database of gene sets used for GSEA (18). |Normalized enrichment score (NES)| ≥ 1, *P* ≤ 0.05, and FDR ≤ 0.25 were selected as the cutoff criteria for statistically significant differences. ES is enrichment score, NES is the normalized ES value after correction, *P* indicates the confidence of enrichment results, and FDR is an estimate of the probability of false-positive results for NES, so the smaller the FDR, the more significant the enrichment (19, 20). The MSigDB gene set includes nine major collections (H:C8). C2 (curated gene sets), C5 (ontology gene sets), and C7 (immunological signature gene sets) were the target datasets for this study. The R code was obtained from https://github.com/dongyihe/rheumatoidarthritis.

### Gene Ontology Functional Enrichment and the Kyoto Encyclopedia of Genes and Genomes Pathway Analysis

The DEGs were annotated using the GO functional enrichment analysis, which included biological process (BP), molecular function (MF), and cellular component (CC) domains, and the KEGG pathway analysis (21). The KEGG is a database that provides information regarding gene functions at the molecular and higher levels, including biochemical pathways (22). Annotation and visualization were performed using the clusterProfiler package (23) (an R package for comparing biological themes among gene clusters). Enrichment analysis was performed using the hypergeometric test. Adjusted *P* < 0.05 was selected as the cutoff criterion indicating a statistically significant difference. *P* was adjusted using FDR.

### PPI Network Construction and Identification of Hub Genes

The protein–protein interaction network was constructed using the Search Tool for the Retrieval of Interacting Genes/Proteins (STRING), a database that provides all the exposed PPI (24). The minimum required interaction score had the highest confidence (0.900). Hub genes were screened and visualized using the Molecular Complex Detection (MCODE) and CytoHubba plugins in Cytoscape version 3.7.2 for the visualization, modeling, and analyses of the molecular and genetic interaction networks (25). The MCODE and CytoHubba plugins can identify hub genes from complex interaction networks and help to lock the hub genes in a computationally efficient manner (26).

### Validation of Hub Gene Expression by Quantitative Real-Time PCR

To validate the reliability of RNA-seq analysis in identifying the DEGs and to determine the expression levels of the 10 selected hub genes, quantitative real-time PCR (qRT-PCR) was conducted. Total RNA was extracted from 9 RA and 15 OA synovial tissue samples using TRIzol Reagent (Thermo Fisher Scientific) and reverse-transcribed to complementary DNA (cDNA) using the PrimeScript™ RT Master Mix (Perfect Real Time) (Takara Bio Incorporation, Beijing, China). A qRT-PCR was then performed using the TB Green® Premix Ex Taq™ (Tli RNase H Plus) (Takara Bio Incorporation). β*-actin* gene was used as the internal reference. The relative messenger RNA (mRNA) expression was calculated using the 2^−**ΔΔ**Ct^ method. The Mann–Whitney *U* test was used for statistical analysis and *P* < 0.05 indicated a significant difference.

## Results

### Identification of the Differentially Expressed Genes in the Rheumatoid Arthritis Synovial Tissue Samples Using RNA Sequencing

**Figure 1:**
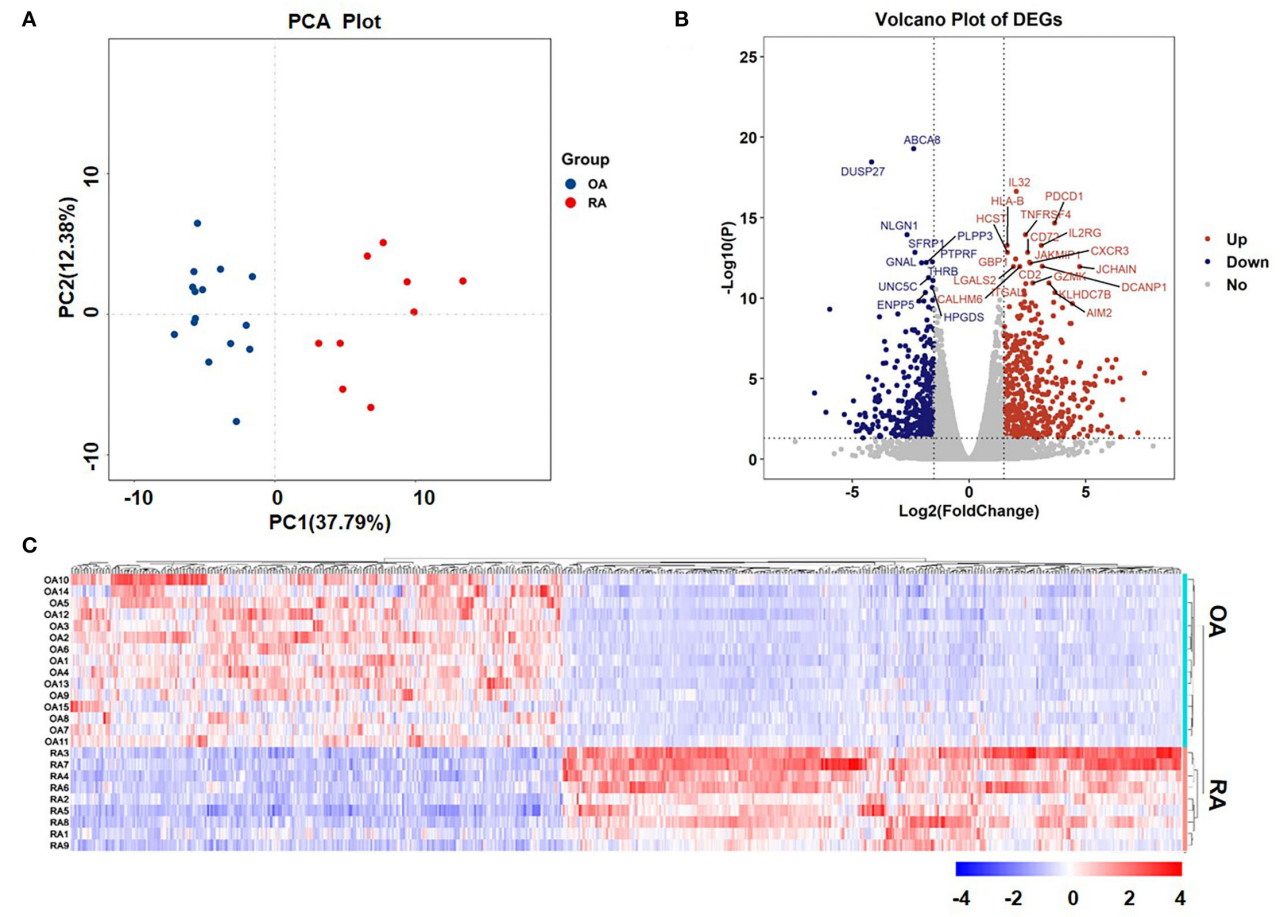
Principal component analysis (PCA) plot of samples and volcano plot and heat map of the differentially expressed genes (DEGs). **(A)** PCA plot of samples and **(B)** Volcano plot of DEGs. The red dots represent upregulated genes and the blue dots represent downregulated genes. The tagged genes are the top 30 genes with a false discovery rate (FDR) < 0.05. **(C)** Heat map of DEGs.

Sequencing data comprised 17,736 genes, of which 851 genes were identified as the DEGs, as they met the following threshold criteria: adjusted *P* < 0.05 and |log2FoldChange| ≥ 1.5. We identified 474 upregulated and 377 downregulated genes. The principal component analysis (PCA) plot of the samples is shown in Figure 1A and the volcano plot and heat map of the DEGs are shown in Figures 1B,C, respectively. Details of the top 30 DEGs are given in Table 2, details of the DEGs encoding chemokines are given in Table 3, and the information about all the DEGs is shown in Supplementary Table 2.

**Table 2:** Information of the top 30 differentially expressed genes (DEGs).

**Gene_id**	**avFPKM_OA**	**avFPKM_RA**	**log2FC**	***p*-Value**	**FDR**
ABCA8	1,946.28	377.46	−2.37	2.73 × 10^−24^	5.15 × 10^−20^
DUSP27	44.94	2.49	−4.18	3.61 × 10^−23^	3.41 × 10^−19^
IL32	408.08	1,658.18	2.02	3.71 × 10^−21^	2.33 × 10^−17^
PDCD1	12.52	159.29	3.67	4.60 × 10^−19^	2.17 × 10^−15^
NLGN1	67.60	10.70	−2.66	3.45 × 10^−18^	1.12 × 10^−14^
TNFRSF4	29.51	157.31	2.41	3.55 × 10^−18^	1.12 × 10^−14^
HLA-B	11,329.54	35,134.52	1.63	2.13 × 10^−17^	5.26 × 10^−14^
IL2RG	144.67	1,236.88	3.10	2.23 × 10^−17^	5.26 × 10^−14^
CD72	41.59	236.99	2.51	7.50 × 10^−17^	1.42 × 10^−13^
HCST	94.90	297.26	1.65	7.34 × 10^−17^	1.42 × 10^−13^
SFRP1	8,919.41	1,780.61	−2.32	8.28 × 10^−17^	1.42 × 10^−13^
GBP1	533.48	2,126.68	2.00	2.37 × 10^−16^	3.72 × 10^−13^
PTPRF	1,963.24	659.86	−1.57	3.85 × 10^−16^	5.59 × 10^−13^
JAKMIP1	7.78	46.89	2.59	4.43 × 10^−16^	5.97 × 10^−13^
PLPP3	7,762.40	2,173.36	−1.84	4.93 × 10^−16^	6.20 × 10^−13^
GNAL	651.95	158.73	−2.04	5.46 × 10^−16^	6.44 × 10^−13^
CXCR3	20.85	127.90	2.62	6.15 × 10^−16^	6.83 × 10^−13^
DCANP1	7.64	67.11	3.14	9.92 × 10^−16^	1.04 × 10^−12^
CALHM6	76.69	344.55	2.17	1.22 × 10^−15^	1.12 × 10^−12^
JCHAIN	347.73	9,237.36	4.73	1.25 × 10^−15^	1.12 × 10^−12^
LGALS2	29.85	111.12	1.90	1.22 × 10^−15^	1.12 × 10^−12^
UNC5C	455.87	136.78	−1.74	6.11 × 10^−15^	5.25 × 10^−12^
THRB	708.52	243.86	−1.54	1.01 × 10^−14^	8.20 × 10^−12^
GZMK	66.52	441.11	2.73	1.60 × 10^−14^	1.16 × 10^−11^
KLHDC7B	6.85	73.53	3.42	1.59 × 10^−14^	1.16 × 10^−11^
CD2	95.86	515.80	2.43	1.81 × 10^−14^	1.27 × 10^−11^
HPGDS	370.10	124.98	−1.57	3.19 × 10^−14^	2.15 × 10^−11^
ITGAL	201.08	1,024.91	2.35	5.80 × 10^−14^	3.65 × 10^−11^
AIM2	17.21	219.82	3.67	7.50 × 10^−14^	4.49 × 10^−11^
NPP5	161.32	44.14	−1.87	7.61 × 10^−14^	4.49 × 10^−11^
GZMA	61.58	319.25	2.37	1.03 × 10^−13^	5.88 × 10^−11^

**Table 3:** Information of the differentially expressed chemokine genes.

**Gene_id**	**avFPKM_OA**	**avFPKM_RA**	**log2FC**	***P*-Value**	**FDR**
CXCR3	20.85	127.90	2.62	6.15 × 10^−16^	6.83 × 10^−13^
CCL5	241.33	1,256.73	2.38	3.53 × 10^−13^	1.75 × 10^−10^
CXCL9	137.86	2,202.23	4.00	1.02 × 10^−12^	3.89 × 10^−10^
CXCR6	12.63	74.57	2.56	9.95 × 10^−13^	3.89 × 10^−10^
CXCL11	10.92	88.53	3.02	1.38 × 10^−10^	2.27 × 10^−08^
CCR5	160.37	643.42	2.00	7.81 × 10^−10^	9.46 × 10^−08^
CXCL10	56.74	599.22	3.40	9.14 × 10^−09^	7.98 × 10^−07^
CXCL13	6.55	1,202.88	7.52	7.83 × 10^−08^	4.56 × 10^−06^
CXCR4	965.34	3,547.62	1.88	1.04 × 10^−06^	3.97 × 10^−05^
CXCR5	3.05	68.30	4.48	3.02 × 10^−06^	9.66 × 10^−05^
CXCL3	32.45	151.69	2.22	2.05 × 10^−05^	4.74 × 10^−04^
CXCL6	77.05	321.63	2.06	3.71 × 10^−05^	7.66 × 10^−04^
CXCL5	3.35	54.10	4.01	6.70 × 10^−05^	1.24 × 10^−03^
CCR7	21.62	193.51	3.16	9.99 × 10^−05^	1.72 × 10^−03^
CCR2	12.06	49.70	2.04	2.02 × 10^−04^	2.99 × 10^−03^
CCR6	13.89	73.30	2.40	5.04 × 10^−04^	6.31 × 10^−03^
CCR4	12.55	61.95	2.30	5.77 × 10^−04^	7.03 × 10^−03^
CCL24	2.70	26.65	3.30	3.44 × 10^−03^	2.84 × 10^−02^
CCL17	2.84	28.75	3.34	3.83 × 10^−03^	3.09 × 10^−02^
CCL18	730.31	5,798.06	2.99	3.90 × 10^−03^	3.13 × 10^−02^
CCL11	0.59	4.00	2.77	5.27 × 10^−03^	3.92 × 10^−02^
CCL25	0.48	3.60	2.92	6.15 × 10^−03^	4.40 × 10^−02^

### Identification of RA-Related Gene Signatures Using GSEA

**Figure 2:**
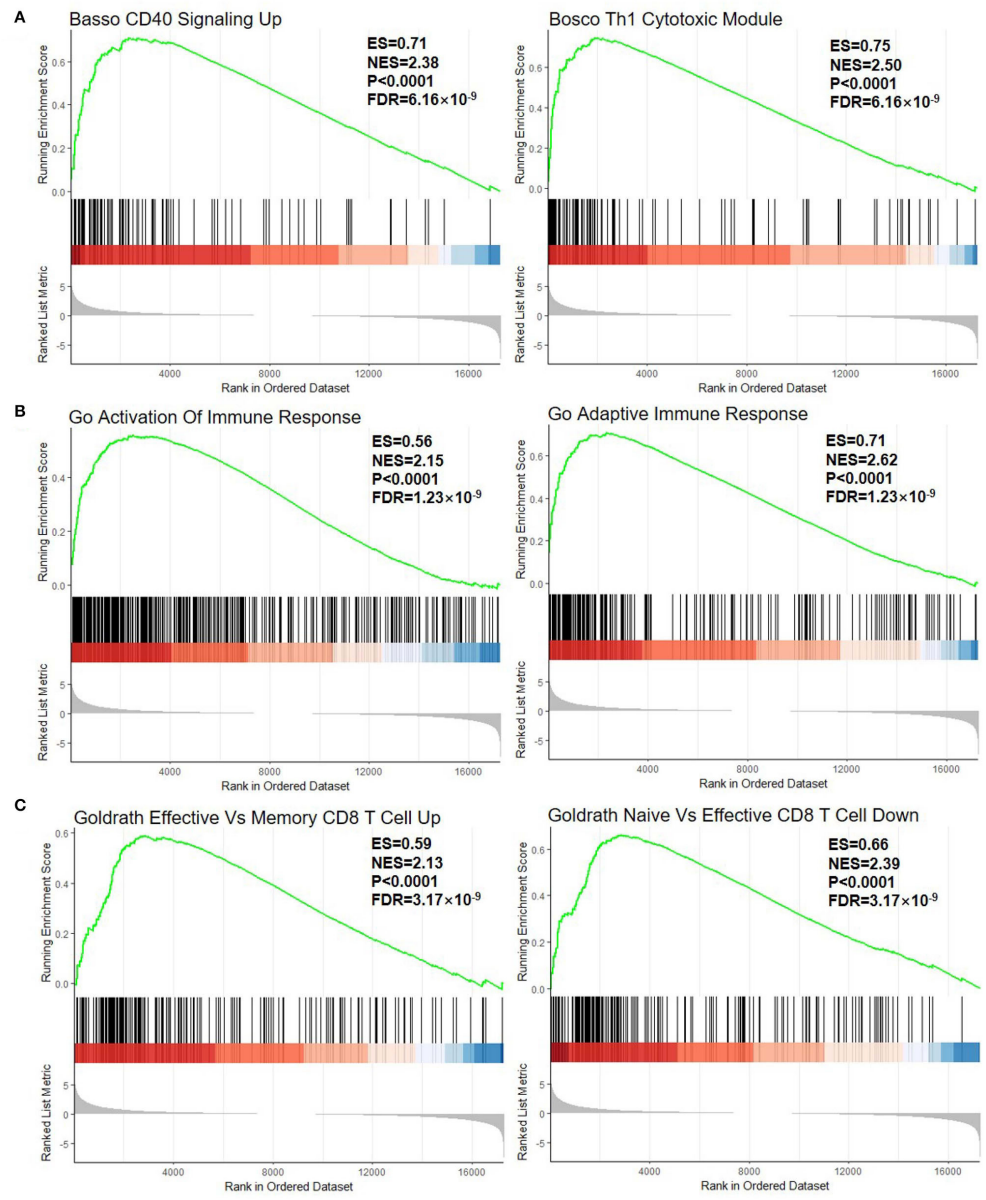
GSEA plot of all the detected genes. **(A)** C2, curated gene sets; **(B)** C5, the Ontology Gene sets; and **(C)** C7, immunologic signature gene sets. GSEA, gene set enrichment analysis; ES, enrichment score; NES, normalized enrichment score. The green line means enrichment profile. The red part of the heat map represents the genes that are highly expressed in rheumatoid arthritis (RA), the blue part of the heat map represents the genes that are highly expressed in osteoarthritis (OA), and the gray area of the heat map represents the signal-to-noise ratio of each gene.

The first part is the enrichment score line graph: the score at the highest peak is the ES value of the gene set. In the second part, the black lines represent gene positions in the sorted gene table. The leading edge subset is the part of the genes corresponding to the origin to the peak ES of the green curve, indicating the genes that have a major contribution to the enrichment. The third part is the distribution of the rank values of all the genes after sorting. The genes corresponding to the red part of the heat map are highly expressed in RA, the genes corresponding to the blue part are highly expressed in OA, and the signal-to-noise ratio corresponding to each gene is shown in the gray area map. In C3 (curated gene sets), the significantly enriched gene sets positively correlated with the RA group were CD40 signaling up (NES = 2.38, FDR = 6.16 × 10^−9^) and Th1 cytotoxic module (NES = 2.50, FDR = 6.16 × 10^−9^) (Figure 2A). In C5 (the Ontology Gene sets), the significantly enriched gene sets positively correlated with the RA group were activation of immune response (NES = 2.15, FDR = 1.23 × 10^−9^) and adaptive immune response (NES = 2.62, FDR = 1.23 × 10^−9^) (Figure 2B). In C7, the upregulation of the gene set of effective vs. memory CD8+ T cell is related to RA-related genes (NES = 2.13, FDR = 3.17 × 10^−9^). The downregulation of the gene set of naïve vs. effective CD8+ T cell is related to RA-related genes (NES = 2.39, FDR = 3.17 × 10^−9^) (Figure 2C).

### Gene Ontology Functional Enrichment Analysis and the Kyoto Encyclopedia of Genes and Genomes Pathway Analysis

**Figure 3:**
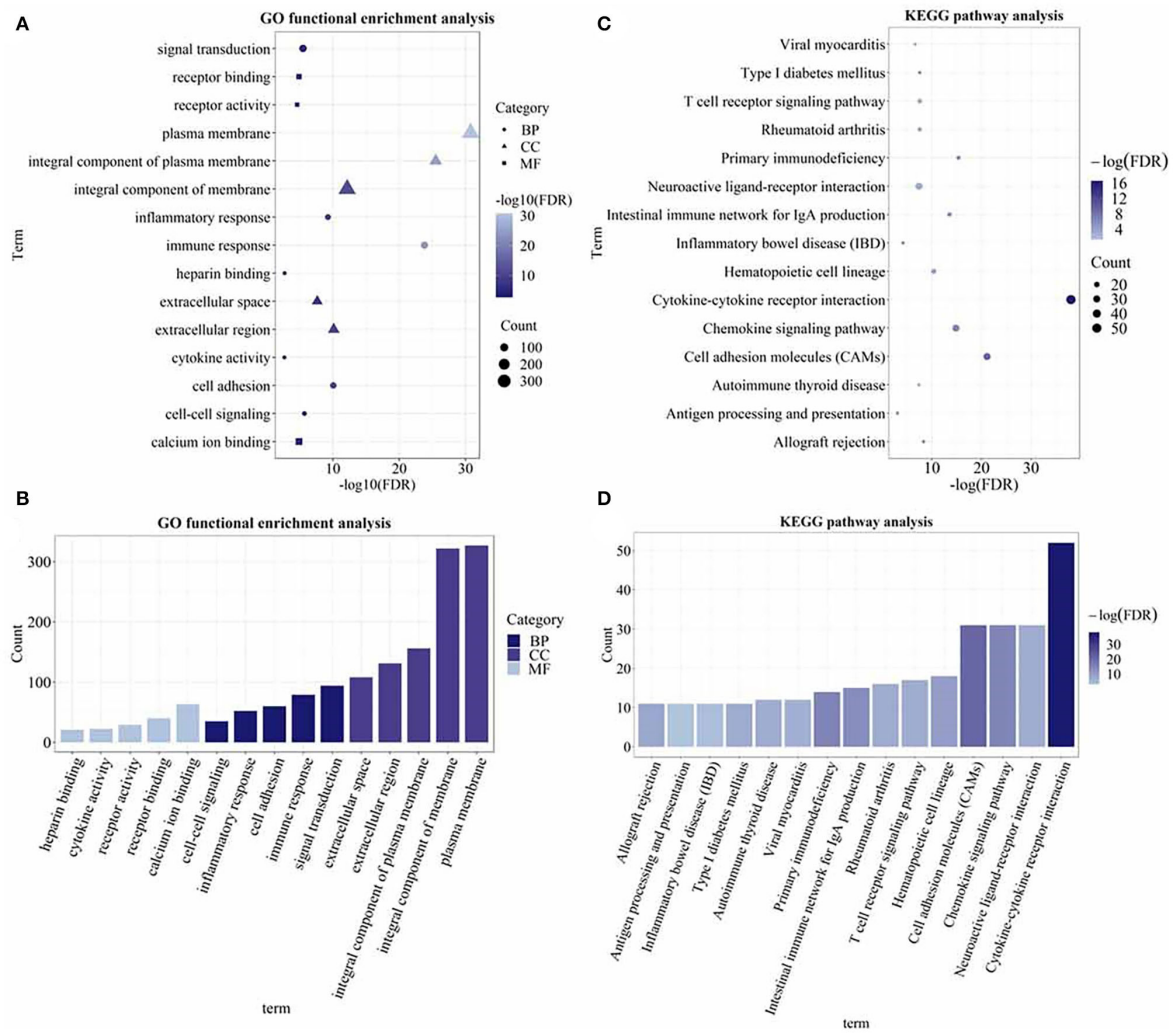
The Gene Ontology (GO) functional enrichment and the Kyoto Encyclopedia of Genes and Genomes (KEGG) pathway analysis of the DEGs. **(A)** Dot plot of the GO functional enrichment analysis (the top five terms of each domain); **(B)** bar plot of the GO functional enrichment analysis (the top five terms of each domain); **(C)** dot plot of the KEGG pathway analysis; and **(D)** bar plot of the KEGG pathway analysis. The size of the dots and the height of the histogram represent the number of enriched genes and the color represents the *P*-value. The bigger the dot, the higher the column, and the more genes are enriched. The darker the color, the smaller the *P*-value.

**Figure 4:**
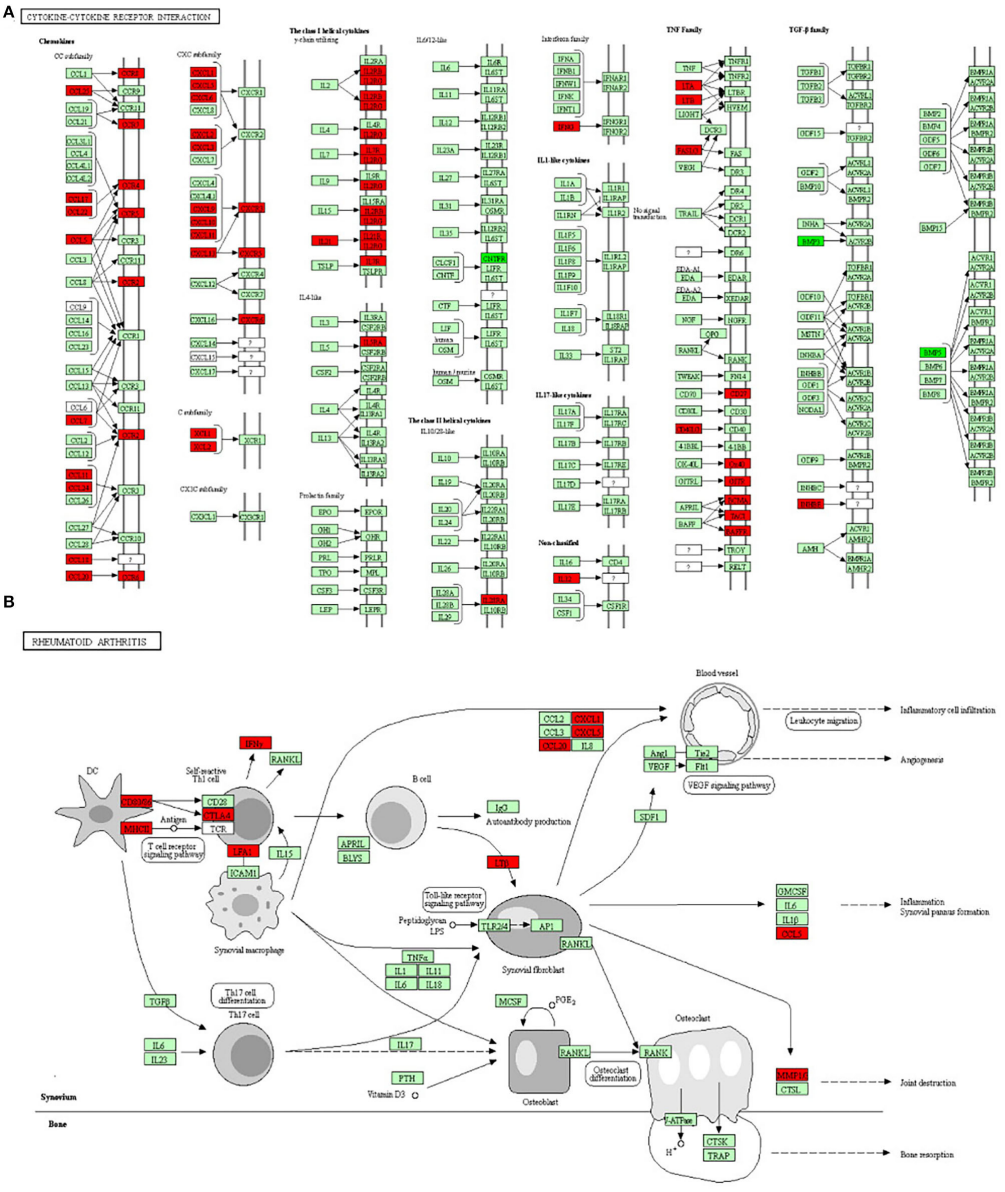
The two most important pathways identified through the KEGG pathway analysis. **(A)** The cytokine–cytokine receptor interaction pathway and **(B)** the rheumatoid arthritis pathway. The red boxes represent upregulated genes and the dark green boxes represent downregulated genes.

The gene ontology functional enrichment analysis revealed that the DEGs were enriched in the signal transduction (*P* = 3.01 × 10^−6^), immune response (*P* = 1.65 × 10^−24^), and inflammatory response (*P* = 5.76 × 10^−10^) functions of the BP domain. In the MF domain, the DEGs were enriched in the calcium ion binding (*P* = 1.26 × 10^−5^), receptor binding (*P* = 1.26 × 10^−5^), and cytokine activity (*P* = 2.01 × 10^−3^) functions. In the CC domain, the DEGs were enriched in the plasma membrane (*P* = 1.91 × 10^−31^), integral component of membrane (*P* = 7.39 × 10^−13^), and extracellular region (*P* = 7.63 × 10^−11^) functions (Figures 3A,B). Detailed information is given in Supplementary Table 3. The KEGG pathway analysis revealed that the DEGs were enriched in the cytokine–cytokine receptor interaction (*P* = 3.05 × 10^−17^), chemokine signaling (*P* = 3.50 × 10^−7^), T-cell receptor signaling (*P* = 5.17 × 10^−4^), and rheumatoid arthritis (*P* = 5.17 × 10^−4^) pathways (Figures 3C,D). The important pathways involved in the cytokine–cytokine receptor interaction and RA are shown in Figure 4. Detailed information is given in Supplementary Table 4.

### PPI Network Development and Identification of Hub Genes in Rheumatoid Arthritis

**Figure 5:**
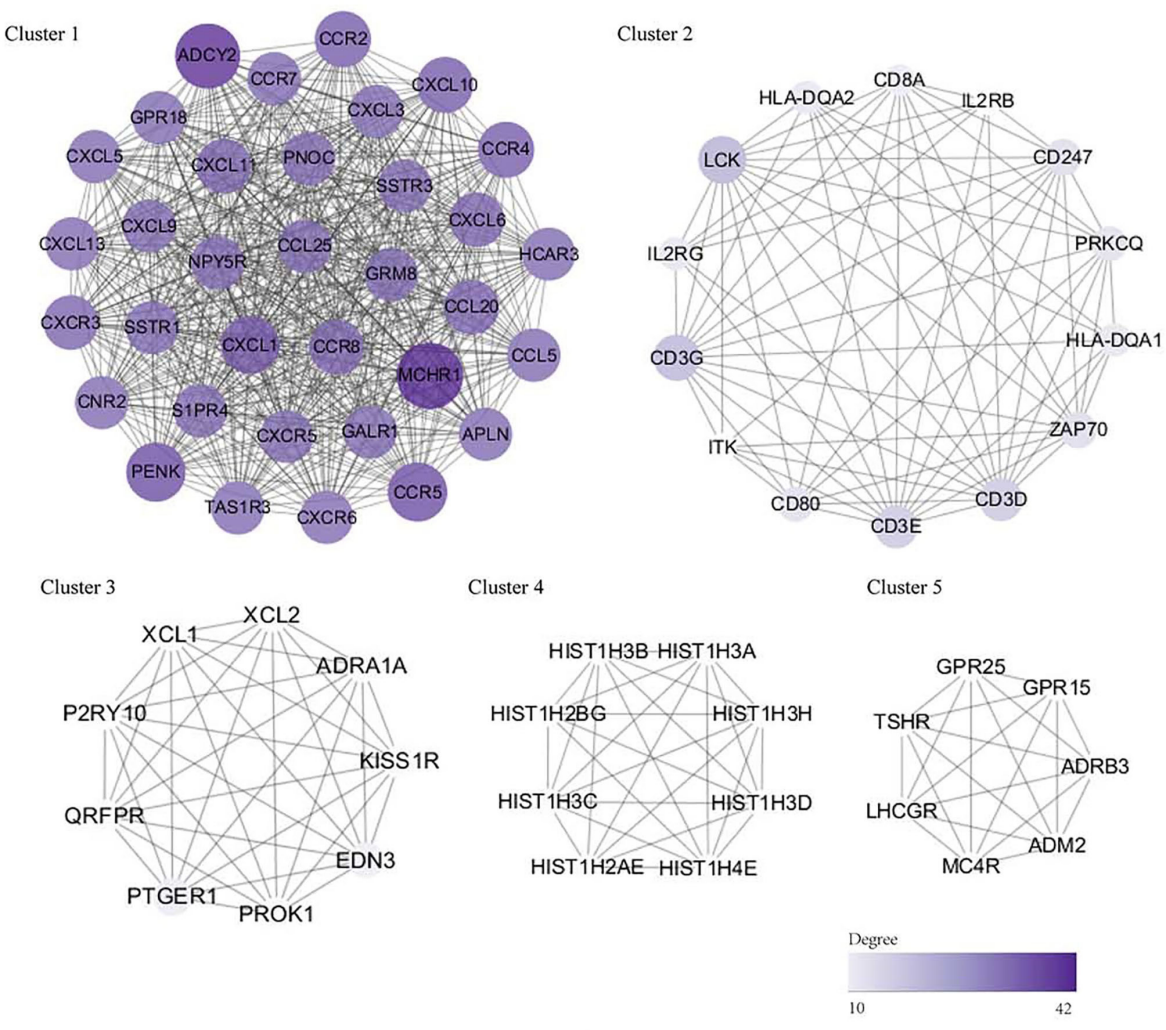
The PPI network and hub genes (top five modules). The significant modules identified by the Molecular Complex Detection (MCODE) and the cytoHubba plugin of Cytoscape. The size and color of circles represent the degree of the hub gene. The darker the color, the bigger the circle, and the higher the degree of the core gene. DEG, differentially expressed gene; PPI, protein–protein interaction.

Using the STRING database, our analysis produced 833 nodes and 1,639 edges and the PPI enrichment *P*-value was 1.0 × 10^−16^. Using the MCODE plugin in Cytoscape, 30 modules were identified. The important five modules are given in Figure 5. Using both the MCODE and cytoHubba plugins in Cytoscape, we identified C-X-C motif chemokine ligand (*CXCL*) 13, *CXCL6, CXCL3, CXCL10*, C-C motif chemokine receptor (*CCR*) *5, CCR2, CCR7*, C-X-C motif chemokine receptor 5 (*CXCR5*), somatostatin receptor (*SSTR*) *1*, and *SSTR3* genes as the hub genes.

### Validation of Rheumatoid Arthritis-Related Hub Genes

**Figure 6:**
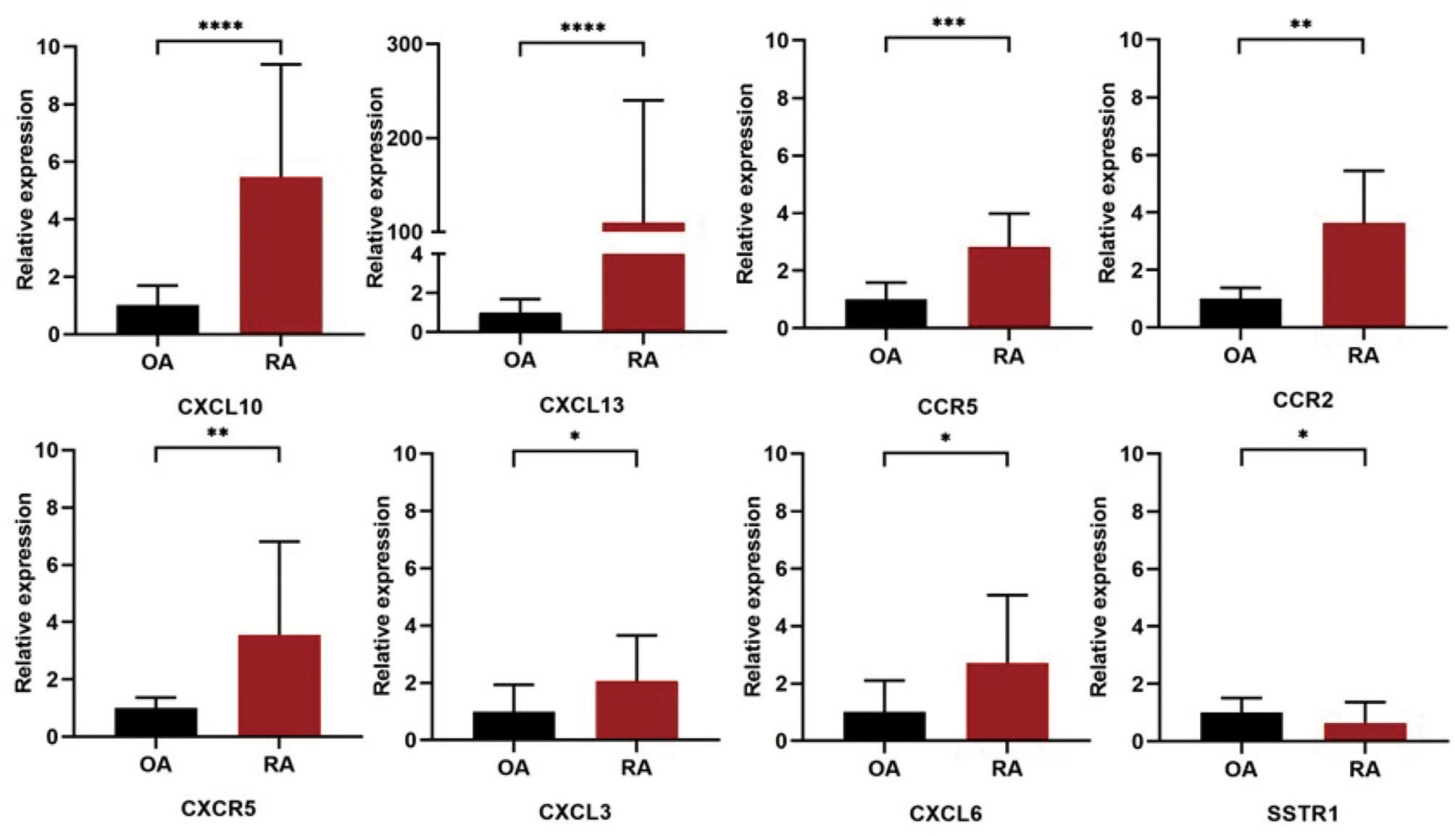
Validation of the 10 hub DEGs identified in the synovial tissue samples of patients with RA and OA by quantitative real-time PCR (qRT-PCR). The relative expression levels of each gene were calculated using the 2^−ΔΔCt^ method. ****, *P* < 0.0001, ***, *P* < 0.005, **, *P* < 0.01, *, *P* < 0.05.

The expression levels of the 10 selected hub genes in the synovial tissue samples from patients with RA and OA were validated by qRT-PCR. The primer sequences used for this experiment are given in Table 4. Statistical analysis showed that the expression levels of *CXCL13* (*P* < 0.0001), *CXCL6* (*P* = 0.0252), *CCR5* (*P* = 0.0002), *CXCR5* (*P* = 0.0033), *CCR2* (*P* = 0.0073), *CXCL3* (*P* = 0.0314), and *CXCL10* (*P* < 0.0001) in the RA synovial tissue samples were significantly higher than those in the OA synovial tissue samples, while the expression level of *SSTR1* (*P* = 0.0486) was significantly higher in the OA synovial tissue samples than in the RA synovial tissue samples (Figure 6).

**Table 4:** Gene primer sequences.

**Gene**	**Primer sequence**	
SSTR3	FORWARD	5′-ATGGACATGCTTCATCCATCAT-3′
	REVERSE	5′-CACATAGATGACCAGCGAGTTA-3′
SSTR1	FORWARD	5′-TGTTGTACACATTTCTCATGGG-3′
	REVERSE	5′-CATCTTAGCAATGATGAGCACG-3′
CCR5	FORWARD	5′-GCAGCTCTCATTTTCCATACAG-3′
	REVERSE	5′- GACACCGAAGCAGAGTTTTTAG-3′
CCR7	FORWARD	5′-CATGCTCCTACTTCTTTGCATC-3′
	REVERSE	5′-CACTGTGGCTAGTATCCAGATG-3′
CXCL6	FORWARD	5′-TGAGAGTAAACCCCAAAACGAT-3′
	REVERSE	5′-CAAACTTGCTTCCCGTTCTTC-3′
CXCL3	FORWARD	5′-GCGTCCGTGGTCACTGAACTG-3′
	REVERSE	5′-AGTGTGGCTATGACTTCGGTTTGG-3′
CCR2	FORWARD	5′-CCAACGAGAGCGGTGAAGAAGTC-3′
	REVERSE	5′- CGAGTAGAGCGGAGGCAGGAG-3′
CXCR5	FORWARD	5′-CGGCAGACACGCAGTTCCAC-3′
	REVERSE	5′-ACGGCAAAGGGCAAGATGAAGAC-3′
CXCL10	FORWARD	5′-CTCTCTCTAGAACTGTACGCTG-3′
	REVERSE	5′-ATTCAGACATCTCTTCTCACCC-3′
CXCL13	FORWARD	5′-CAAGGTGTTCTGGAGGTCTATT-3′
	REVERSE	5′-TGAATTCGATCAATGAAGCGTC-3′

## Discussion

Rheumatoid arthritis and OA are two common types of arthritis that present with inflammation but have distinct etiologies, clinical trajectories, and treatments. The pathogenesis and manifestations of these two diseases are complex, with clinical heterogeneity in presentation and disease course (8, 27). Distinguishing between RA and OA is critically important for early diagnosis, appropriate treatment, and elucidation of the underlying pathophysiology of these disorders. Previous studies have demonstrated that synovial tissue plays an important role in the occurrence and development of RA and OA. Previous studies mostly used microarray technology to study the differential expression profile data of RA and OA, which played an important role in the study of RA. However, microarray technology can only detect known sequences, while sequencing technology can detect unknown sequences and discover unknown genes. Compared with first-generation sequencing technology, second-generation sequencing technology has high throughput and high sensitivity and may discover new disease-causing genes. In this study, second-generation sequencing was used to analyze the synovial tissue samples obtained from patients with RA and OA. We identified the DEGs between the two samples, analyzed the functions and pathways of the DEGs, and validated the hub DEGs by qRT-PCR.

In previous studies, synovial tissue datasets in the Gene Expression Omnibus (GEO) database, such as GSE55235 and GSE12021, were used for bioinformatics analysis of RA. These studies were based on different datasets and identified genes (28, 29). Li et al. (30) found that vascular endothelial growth factor A and epidermal growth factor receptor may have essential roles in the development of RA and can be used as potential biomarkers of RA. Ren et al. (29) suggested that a set of eight genes (*CCR5, CCL5, CXCL9, CXCL10, CXCL13, PNOC, TLR8*, and *CD52*) can be used to diagnose RA with excellent specificity and sensitivity. To further analyze the transcriptome of the synovial tissue samples of patients with RA, we collected the samples from patients with RA and OA present at the same rheumatology hospital, performed RNA-seq, and investigated the pathways, gene networks, and hub genes. In this study, OA was used as the control group to study RA biomarkers. We performed transcriptomic analysis of the synovial tissue samples of patients with RA and OA using RNA-seq and determined that the identified DEGs can be used as biomarkers for diagnosing the two diseases. Further study using these biomarkers should be conducted.

In this study, 17,736 genes were identified. GSEA is sensitive in detecting genes with relatively small fold changes (31). The significantly enriched curated gene sets that positively correlated with RA were CD40 signaling and Th1 cytotoxic module. CD40 signaling is associated with the production of human rheumatoid factor (32) and the CD40/nuclear factor-kappa B (NF-kB) signaling pathway plays an important role in RA pathogenesis (33). The Th1 cytotoxic module has not been reported to be related to RA, but Th1 cytotoxicity is reportedly associated with the tumor microenvironment (34). The significantly enriched Ontology Gene sets that positively correlated with the RA group were involved in the activation of the immune response and adaptive immune response. RA is an autoimmune disease that affects both innate and adaptive immunities (35). The significantly enriched immunologic signature gene sets that positively correlated with the RA group were effective vs. memory CD8+ T cells (upregulated) and naïve vs. effective CD8+ T cells (downregulated). CD8+ T cells are involved in the pathogenesis of many autoimmune diseases, mainly because of their self-reactive cytotoxic inflammatory behavior (36). Effective CD8+ T cells have proliferative and cytotoxic properties and induce the death of infected cells and effective memory CD8+ T cells have a lower ability to induce cytotoxicity than effective CD8+ T cells (36, 37).

In this study, 851 DEGs were identified, of which 474 DEGs were upregulated and 377 DEGs were downregulated. The GO functional enrichment analysis revealed that the DEGs were enriched in signal transduction, immune response, and inflammatory response (BP domain); in calcium ion binding, receptor binding, and chemokine activity (MF domain); and in the plasma membrane, an integral component of membrane, and extracellular region (CC domain). The KEGG pathway analysis showed that the DEGs were enriched in the cytokine–cytokine receptor interactions, chemokine signaling, T-cell receptor signaling, and RA pathways. The DEGs were mainly concentrated in immune and inflammation-related pathways.

Ten DEGs were identified as hub genes using the MCODE and the cytoHubba plugin of Cytoscape. According to the qRT-PCR validation, the expression levels of *CXCL13, CXCL6, CCR5, CXCR5, CCR2, CXCL3*, and *CXCL10* in the RA synovial tissue samples were higher than that in OA synovial tissue samples, while the expression of *SSTR1* showed the opposite trend. The expression levels of *CCR7* and *SSTR3* did not differ between the RA and OA synovial tissue samples. *CXCL13, CXCL10, CXCL6*, and *CXCL3* are the main members of the chemokine subfamily CXC. *CXCL13*, a B-cell chemokine, interacts with its receptor *CXCR5* to promote the migration and aggregation of B lymphocytes (38). The expression level of *CXCL13* in the serum of patients with RA is positively correlated with the level of rheumatoid factor and with disease activity and treatment response in early RA (39–41). *CXCL10* is a ligand for *CXCR3*, which may stimulate the migration of monocytes, natural killer cells, and T cells (42). The expression of *CXCL10* has been detected in the serum, synovial fluid, and synovial tissue of patients with RA (43, 44). Therefore, *CXCL10* could act as a disease activity marker in early RA because of its high level in the plasma of untreated early patients with RA and its association with clinical disease activity (45). This study confirmed the high expression level of *CXCL10* in the RA synovial tissue samples, which was significantly higher than that in the OA synovial tissue samples. *CXCL3* is associated with the invasion and metastasis of various cancers (46–48) and *CXCL3* and *CXCL6* are involved in the invasion and migration of various cancers (49–51). The differential expression of *CXCL3* and *CXCL6* in the RA and OA synovial tissue samples is not yet reported. *CCR7, CCR5*, and *CCR2* are chemokine receptors. *CCR5* is expressed in RA synovial tissue and in T-helper cell type 1 inflammatory infiltrates. The Delta32 allelic variant of *CCR5* has been reported to have a protective effect on RA susceptibility (52); however, the effect of *CCR5* inhibitors on RA remains controversial (53–55). *CCR2* has been widely considered as a potential therapeutic target for RA and *CCR2* blocking agents have been developed (56). Monocyte chemoattractant protein 1 (*CCL2*) and its high-affinity receptor *CCR2* are central to the development of pain associated with knee OA. Thus, *CCR2* plays an important role in both the RA and OA. This study found that the expression levels of *CCR2* in the RA and OA synovial tissue samples were different, which are likely related to its different functions in RA and OA pathogenesis. Somatostatins can regulate diverse cellular functions such as neurotransmission and cell proliferation. *SSTR1* is associated with various cancers, such as prostate cancer (57) and gastric cancer (58). The role of *SSTR1* in RA and OA has not yet been studied and the present results may provide a basis for future study on arthritis.

Osteoarthritis referred to degenerative joint disease and RA referred to joint disease caused by immune disorders. OA was used as the control group for RA, which had advantages but also limitations. The main limitation of this study was that inflammation was not properly investigated. RA is characterized by persistent synovitis and systemic inflammation and in the course of the development of OA, synovial inflammation is also observed. Although study on systemic inflammation in OA remains controversial, RA and OA have different mechanisms of inflammation as elucidated by the present results. These different mechanisms will be the focus of our future study.

In this study, RNA-seq technology was used to supplement the previous microarray technology and qRT-PCR technology was used to supplement and verify the previous conclusions. At the same time, bioinformatics technology was combined with experimental technology to make the results more reliable and provide a reliable preliminary basis for future study.

## Conclusion

Ribonucleic acid sequencing was used to detect differential gene expression in the RA and OA synovial tissue samples. Using bioinformatics, the DEGs were identified in the RA and OA synovial tissue samples and the GO functional and the KEGG pathway enrichment analyses of the DEGs were performed. The hub DEGs such as *SSTR1, CXCR5, CXCL6, CXCL3, CXCL13, CXCL10, CCR7*, and *CCR2* were validated by qRT-PCR. This study enriched the expression profile data of the DEGs in the synovial tissue of patients with RA and OA and provides novel insights into the differences between RA and OA. The candidate DEG pathways might be therapeutic targets and biomarkers for RA or OA.

## Data Availability Statement

The datasets presented in this study can be found in online repositories. The names of the repository/repositories and accession number(s) can be found at: https://github.com/dongyihe/rheumatoidarthritis.

## Ethics Statement

The studies involving human participants were reviewed and approved by Shanghai Guanghua Hospital. The patients/participants provided their written informed consent to participate in this study.

## Author Contributions

DH and SG contributed to the conception, design, and final approval of the manuscript. CC, LX, and YB contributed to sequencing data and statistical analyses. YSh, YSu, SS, and SJS collected the samples, helped with statistical analysis, and drafted the manuscript. The final manuscript was written by RZ and YJ. All the authors have read and approved the final version of the manuscript.

## Funding

This work was funded by the National Natural Science Funds of China (82074234 and 82004166); Shanghai Chinese Medicine Development Office, National Administration of Traditional Chinese Medicine, Regional Chinese Medicine (Specialist) Diagnosis and Treatment Center Construction Project-Rheumatology; State Administration of Traditional Chinese Medicine, National TCM Evidence-Based Medicine Research and Construction Project, Basic TCM Evidence-Based Capacity Development Program; Shanghai Municipal Health Commission, East China Region based Chinese and Western Medicine Joint Disease Specialist Alliance; National Key Research and Development Project (2018YFC1705203).

## Conflict of Interest

The authors declare that the research was conducted in the absence of any commercial or financial relationships that could be construed as a potential conflict of interest.

## Publisher's Note

All claims expressed in this article are solely those of the authors and do not necessarily represent those of their affiliated organizations, or those of the publisher, the editors and the reviewers. Any product that may be evaluated in this article, or claim that may be made by its manufacturer, is not guaranteed or endorsed by the publisher.
