# Supplementary material for: RNA-seq and Network Analysis Reveal Unique Chemokine Activity Signatures in the Synovial Tissue of Patients With Rheumatoid Arthritis
Source: Front Med (Lausanne). 2022 May 4;9:799440. doi: 10.3389/fmed.2022.799440 (PMC9116426; doi:10.3389/fmed.2022.799440)
Supplement: Supplementary file 1 [file Data_Sheet_1.docx]

**Supplementary Table1 The Statistics of Clinical Information of RA and OA Patients**

|  | RA | OA | P |
| --- | --- | --- | --- |
| Gender  Age | 9 (8 female/1male)  70 (73,62) | 15(female)  74 (75,68) | 0.19  0.07 |
| Course of disease | 10 (30,5) | 10 (13,5) | 0.33 |
| ESR | 53 (66,34) | 12 (17,5) | 0.0009 |
| CRP | 19.6 (58.4,5.7) | 0.62 (1.82,0.50) | 0.0012 |

**Supplementary Table2 The Information of all DEGs**

| Gene_id | BaseMean  OA | BaseMean  RA | log2FC | pValue | FDR |
| --- | --- | --- | --- | --- | --- |
| ABCA8 | 1946.28 | 377.46 | -2.37 | 2.73E-24 | 5.15E-20 |
| ABCC6 | 182.05 | 60.49 | -1.59 | 2.54E-04 | 3.61E-03 |
| ACADL | 28.31 | 3.89 | -2.86 | 1.80E-03 | 1.72E-02 |
| ACAP1 | 104.74 | 671.59 | 2.68 | 9.31E-12 | 2.41E-09 |
| ACHE | 16.85 | 64.52 | 1.94 | 2.68E-04 | 3.78E-03 |
| ACOXL | 2.72 | 45.55 | 4.06 | 2.91E-03 | 2.50E-02 |
| ACSL6 | 11.75 | 37.45 | 1.67 | 6.23E-04 | 7.46E-03 |
| ACTA1 | 2.85 | 11.68 | 2.04 | 1.61E-04 | 2.49E-03 |
| ACTR3C | 12.38 | 39.04 | 1.66 | 7.56E-04 | 8.70E-03 |
| ACY3 | 1.41 | 8.96 | 2.67 | 1.99E-04 | 2.96E-03 |
| ADAM12 | 420.10 | 1741.19 | 2.05 | 9.54E-04 | 1.05E-02 |
| ADAM19 | 271.58 | 775.05 | 1.51 | 1.26E-10 | 2.15E-08 |
| ADAM22 | 444.37 | 107.85 | -2.04 | 2.42E-10 | 3.65E-08 |
| ADAM28 | 148.44 | 685.90 | 2.21 | 3.24E-10 | 4.66E-08 |
| ADAM8 | 144.49 | 754.70 | 2.38 | 2.14E-05 | 4.90E-04 |
| ADAMDEC1 | 28.35 | 611.23 | 4.43 | 4.95E-13 | 2.23E-10 |
| ADAMTS19 | 49.56 | 13.48 | -1.88 | 1.90E-03 | 1.80E-02 |
| ADAMTSL2 | 850.95 | 291.11 | -1.55 | 6.86E-06 | 1.93E-04 |
| ADCY2 | 120.73 | 29.00 | -2.06 | 4.16E-03 | 3.28E-02 |
| ADCY5 | 208.67 | 56.16 | -1.89 | 1.03E-07 | 5.81E-06 |
| ADCY8 | 38.75 | 10.49 | -1.88 | 2.16E-03 | 1.99E-02 |
| ADGRE2 | 104.44 | 317.72 | 1.61 | 2.45E-07 | 1.18E-05 |
| ADGRG5 | 9.57 | 96.20 | 3.33 | 3.52E-07 | 1.61E-05 |
| ADH1C | 200.37 | 45.60 | -2.14 | 4.98E-03 | 3.75E-02 |
| ADIPOQ | 1280.41 | 66.76 | -4.26 | 2.36E-03 | 2.13E-02 |
| ADM2 | 2.00 | 13.74 | 2.78 | 1.97E-04 | 2.94E-03 |
| ADORA2A | 53.72 | 167.34 | 1.64 | 1.17E-06 | 4.34E-05 |
| ADRA1A | 38.11 | 8.97 | -2.09 | 5.39E-07 | 2.27E-05 |
| AIM2 | 17.21 | 219.82 | 3.67 | 7.50E-14 | 4.49E-11 |
| AKR1B15 | 83.49 | 6.37 | -3.71 | 6.43E-07 | 2.62E-05 |
| ALPK2 | 35.58 | 113.05 | 1.67 | 1.41E-03 | 1.42E-02 |
| ALX4 | 78.07 | 12.95 | -2.59 | 1.42E-07 | 7.57E-06 |
| AMPD1 | 2.08 | 52.62 | 4.66 | 2.20E-05 | 5.02E-04 |
| ANGPTL7 | 2032.99 | 229.61 | -3.15 | 4.94E-03 | 3.73E-02 |
| ANKFN1 | 10.73 | 1.73 | -2.63 | 1.41E-05 | 3.48E-04 |
| ANKRD22 | 7.09 | 61.42 | 3.12 | 4.71E-08 | 2.99E-06 |
| ANO3 | 14.13 | 1.73 | -3.03 | 8.73E-06 | 2.35E-04 |
| ANO4 | 9.27 | 2.72 | -1.77 | 4.88E-03 | 3.70E-02 |
| ANO9 | 34.01 | 252.76 | 2.89 | 2.99E-07 | 1.40E-05 |
| ANXA8L1 | 5.65 | 0.38 | -3.91 | 3.44E-04 | 4.63E-03 |
| AP3B2 | 18.76 | 5.62 | -1.74 | 3.70E-04 | 4.91E-03 |
| APLN | 129.20 | 615.68 | 2.25 | 2.28E-03 | 2.08E-02 |
| APOBEC3B | 12.58 | 43.01 | 1.77 | 4.51E-07 | 1.96E-05 |
| APOBEC3H | 10.74 | 40.77 | 1.93 | 1.60E-09 | 1.79E-07 |
| AQP7 | 207.60 | 45.43 | -2.19 | 2.26E-03 | 2.06E-02 |
| AQP9 | 54.26 | 208.17 | 1.94 | 2.47E-05 | 5.57E-04 |
| AR | 545.45 | 164.23 | -1.73 | 8.83E-13 | 3.62E-10 |
| ARMC4 | 5.46 | 1.04 | -2.40 | 4.15E-03 | 3.28E-02 |
| ARRDC5 | 5.00 | 14.51 | 1.54 | 1.55E-03 | 1.52E-02 |
| ARX | 0.38 | 6.23 | 4.03 | 5.30E-03 | 3.94E-02 |
| ASCL2 | 21.62 | 66.28 | 1.62 | 2.44E-08 | 1.77E-06 |
| ASXL3 | 9.79 | 2.26 | -2.11 | 3.03E-03 | 2.58E-02 |
| ATP1A2 | 502.64 | 131.06 | -1.94 | 2.59E-05 | 5.79E-04 |
| ATP1A3 | 3.88 | 19.24 | 2.31 | 1.78E-07 | 9.16E-06 |
| ATRNL1 | 21.73 | 5.80 | -1.90 | 2.77E-04 | 3.85E-03 |
| B3GALT1 | 21.70 | 3.87 | -2.49 | 6.58E-05 | 1.22E-03 |
| B3GAT1 | 7.96 | 38.89 | 2.29 | 2.38E-06 | 7.92E-05 |
| BATF | 41.08 | 173.51 | 2.08 | 4.37E-09 | 4.26E-07 |
| BCL11A | 9.75 | 60.47 | 2.63 | 1.26E-08 | 1.03E-06 |
| BCL11B | 35.58 | 202.12 | 2.51 | 3.72E-08 | 2.49E-06 |
| BCL2A1 | 27.45 | 214.12 | 2.96 | 3.15E-12 | 9.92E-10 |
| BCL2L14 | 0.88 | 7.72 | 3.13 | 3.17E-04 | 4.32E-03 |
| BEGAIN | 375.09 | 102.72 | -1.87 | 9.68E-05 | 1.68E-03 |
| BFSP2 | 0.41 | 8.29 | 4.35 | 6.06E-04 | 7.29E-03 |
| BHLHA15 | 2.08 | 69.77 | 5.07 | 4.55E-03 | 3.50E-02 |
| BICDL1 | 21.86 | 157.40 | 2.85 | 7.43E-13 | 3.18E-10 |
| BIK | 3.91 | 19.50 | 2.32 | 3.06E-04 | 4.21E-03 |
| BIRC3 | 344.41 | 1301.87 | 1.92 | 8.01E-04 | 9.11E-03 |
| BLK | 7.64 | 150.94 | 4.30 | 2.70E-04 | 3.79E-03 |
| BMP5 | 314.11 | 37.72 | -3.06 | 2.98E-12 | 9.67E-10 |
| BMPR1B | 69.68 | 21.29 | -1.71 | 1.32E-05 | 3.30E-04 |
| BRINP1 | 42.89 | 6.45 | -2.73 | 6.88E-05 | 1.26E-03 |
| BRSK2 | 9.62 | 3.19 | -1.59 | 4.69E-03 | 3.59E-02 |
| BTC | 335.62 | 89.71 | -1.90 | 3.78E-09 | 3.84E-07 |
| BTLA | 7.53 | 120.51 | 4.00 | 7.94E-07 | 3.13E-05 |
| BTN1A1 | 0.44 | 4.02 | 3.18 | 2.50E-03 | 2.23E-02 |
| C10orf105 | 2516.14 | 857.37 | -1.55 | 1.19E-05 | 3.03E-04 |
| C10orf82 | 4.58 | 0.80 | -2.52 | 6.74E-03 | 4.72E-02 |
| C11orf21 | 56.42 | 165.52 | 1.55 | 5.33E-10 | 6.94E-08 |
| C11orf87 | 16.43 | 0.80 | -4.36 | 1.37E-03 | 1.39E-02 |
| C12orf42 | 0.57 | 6.94 | 3.59 | 3.73E-04 | 4.95E-03 |
| C14orf180 | 54.30 | 5.07 | -3.42 | 5.49E-05 | 1.06E-03 |
| C15orf48 | 10.08 | 229.76 | 4.51 | 6.82E-06 | 1.92E-04 |
| C16orf54 | 95.09 | 297.55 | 1.65 | 2.35E-10 | 3.58E-08 |
| C16orf74 | 9.62 | 38.53 | 2.00 | 2.70E-04 | 3.79E-03 |
| C19orf38 | 21.75 | 66.60 | 1.61 | 9.46E-10 | 1.12E-07 |
| C1orf226 | 60.07 | 16.01 | -1.91 | 7.82E-10 | 9.46E-08 |
| C1QTNF8 | 2.50 | 0.11 | -4.54 | 7.00E-03 | 4.85E-02 |
| C2orf40 | 1542.03 | 415.02 | -1.89 | 5.16E-08 | 3.21E-06 |
| C4orf50 | 2.19 | 16.41 | 2.90 | 4.10E-04 | 5.33E-03 |
| C6 | 534.25 | 49.01 | -3.45 | 1.91E-06 | 6.57E-05 |
| C7 | 3072.30 | 363.20 | -3.08 | 2.73E-04 | 3.82E-03 |
| CACNA1G | 56.87 | 16.11 | -1.82 | 2.11E-03 | 1.96E-02 |
| CACNG6 | 1.08 | 6.91 | 2.67 | 4.67E-03 | 3.57E-02 |
| CADM2 | 7.25 | 1.94 | -1.90 | 5.01E-03 | 3.77E-02 |
| CALB2 | 43.41 | 11.13 | -1.96 | 6.75E-03 | 4.72E-02 |
| CALHM6 | 76.69 | 344.55 | 2.17 | 1.22E-15 | 1.12E-12 |
| CAMK1G | 1.46 | 6.00 | 2.04 | 6.48E-03 | 4.59E-02 |
| CAMK4 | 33.96 | 114.58 | 1.75 | 1.49E-04 | 2.34E-03 |
| CAMSAP3 | 2.24 | 9.35 | 2.06 | 5.50E-03 | 4.05E-02 |
| CARD17 | 1.78 | 8.95 | 2.33 | 2.41E-04 | 3.47E-03 |
| CARMIL2 | 32.42 | 285.52 | 3.14 | 2.27E-07 | 1.13E-05 |
| CASP5 | 2.67 | 12.97 | 2.28 | 5.35E-04 | 6.63E-03 |
| CCBE1 | 24.29 | 184.21 | 2.92 | 8.66E-10 | 1.03E-07 |
| CCDC158 | 25.42 | 6.61 | -1.94 | 5.95E-07 | 2.47E-05 |
| CCDC78 | 8.09 | 26.78 | 1.73 | 6.61E-03 | 4.66E-02 |
| CCDC85A | 343.30 | 67.02 | -2.36 | 4.93E-11 | 1.02E-08 |
| CCL11 | 0.59 | 4.00 | 2.77 | 5.27E-03 | 3.92E-02 |
| CCL17 | 2.84 | 28.75 | 3.34 | 3.83E-03 | 3.09E-02 |
| CCL18 | 730.31 | 5798.06 | 2.99 | 3.90E-03 | 3.13E-02 |
| CCL22 | 12.06 | 49.70 | 2.04 | 2.02E-04 | 2.99E-03 |
| CCL24 | 2.70 | 26.65 | 3.30 | 3.44E-03 | 2.84E-02 |
| CCL25 | 0.48 | 3.60 | 2.92 | 6.15E-03 | 4.40E-02 |
| CCL5 | 241.33 | 1256.73 | 2.38 | 3.53E-13 | 1.75E-10 |
| CCR2 | 77.05 | 321.63 | 2.06 | 3.71E-05 | 7.66E-04 |
| CCR4 | 12.55 | 61.95 | 2.30 | 5.77E-04 | 7.03E-03 |
| CCR5 | 160.37 | 643.42 | 2.00 | 7.81E-10 | 9.46E-08 |
| CCR6 | 13.89 | 73.30 | 2.40 | 5.04E-04 | 6.31E-03 |
| CCR7 | 21.62 | 193.51 | 3.16 | 9.99E-05 | 1.72E-03 |
| CD160 | 3.92 | 12.44 | 1.67 | 1.44E-03 | 1.44E-02 |
| CD19 | 6.25 | 210.15 | 5.07 | 4.38E-07 | 1.92E-05 |
| CD1B | 1.94 | 9.52 | 2.29 | 1.05E-04 | 1.79E-03 |
| CD2 | 95.86 | 515.80 | 2.43 | 1.81E-14 | 1.27E-11 |
| CD22 | 52.65 | 261.69 | 2.31 | 9.77E-04 | 1.06E-02 |
| CD226 | 51.97 | 149.50 | 1.52 | 2.60E-05 | 5.81E-04 |
| CD247 | 46.07 | 239.65 | 2.38 | 1.05E-12 | 3.89E-10 |
| CD27 | 27.82 | 440.20 | 3.98 | 1.63E-05 | 3.94E-04 |
| CD300E | 319.30 | 950.21 | 1.57 | 7.95E-04 | 9.06E-03 |
| CD38 | 205.10 | 599.13 | 1.55 | 5.12E-05 | 1.00E-03 |
| CD3D | 34.98 | 298.28 | 3.09 | 1.34E-11 | 3.34E-09 |
| CD3E | 106.60 | 687.70 | 2.69 | 4.18E-13 | 1.97E-10 |
| CD3G | 15.68 | 83.50 | 2.41 | 1.22E-07 | 6.69E-06 |
| CD40LG | 14.83 | 83.89 | 2.50 | 7.92E-05 | 1.43E-03 |
| CD48 | 114.50 | 575.33 | 2.33 | 8.28E-11 | 1.52E-08 |
| CD5 | 40.01 | 237.77 | 2.57 | 1.38E-06 | 5.01E-05 |
| CD52 | 142.63 | 858.76 | 2.59 | 9.22E-11 | 1.64E-08 |
| CD6 | 75.41 | 345.00 | 2.19 | 7.35E-08 | 4.31E-06 |
| CD7 | 32.07 | 203.91 | 2.67 | 4.41E-13 | 2.03E-10 |
| CD72 | 41.59 | 236.99 | 2.51 | 7.50E-17 | 1.42E-13 |
| CD79A | 25.48 | 1523.06 | 5.90 | 2.86E-07 | 1.35E-05 |
| CD79B | 117.44 | 377.20 | 1.68 | 2.18E-04 | 3.19E-03 |
| CD80 | 7.99 | 38.14 | 2.26 | 7.29E-06 | 2.02E-04 |
| CD8A | 92.68 | 432.90 | 2.22 | 3.95E-12 | 1.22E-09 |
| CD8B | 39.50 | 113.95 | 1.53 | 4.53E-08 | 2.91E-06 |
| CD96 | 52.86 | 273.20 | 2.37 | 1.25E-08 | 1.03E-06 |
| CDH19 | 121.05 | 24.84 | -2.28 | 1.78E-05 | 4.23E-04 |
| CDH2 | 105.43 | 19.86 | -2.41 | 8.42E-06 | 2.29E-04 |
| CDHR1 | 5.06 | 14.50 | 1.52 | 1.57E-03 | 1.54E-02 |
| CDK5R1 | 24.02 | 70.63 | 1.56 | 1.29E-06 | 4.72E-05 |
| CDO1 | 2929.18 | 950.04 | -1.62 | 5.18E-05 | 1.01E-03 |
| CDON | 5212.07 | 1798.57 | -1.54 | 1.08E-07 | 5.99E-06 |
| CEACAM21 | 28.78 | 106.11 | 1.88 | 6.03E-07 | 2.49E-05 |
| CFB | 3448.00 | 10826.86 | 1.65 | 1.04E-03 | 1.12E-02 |
| CFD | 16234.10 | 5441.04 | -1.58 | 2.71E-08 | 1.93E-06 |
| CHL1 | 144.68 | 37.07 | -1.96 | 5.99E-06 | 1.71E-04 |
| CHRDL1 | 1803.57 | 544.01 | -1.73 | 1.75E-05 | 4.17E-04 |
| CHRNA4 | 8.86 | 0.13 | -6.13 | 6.61E-05 | 1.23E-03 |
| CHST9 | 7.37 | 0.34 | -4.45 | 8.82E-04 | 9.86E-03 |
| CIDEA | 63.36 | 2.72 | -4.54 | 5.87E-04 | 7.11E-03 |
| CIDEC | 228.39 | 10.60 | -4.43 | 1.45E-04 | 2.30E-03 |
| CILP | 8656.32 | 1737.50 | -2.32 | 4.27E-05 | 8.62E-04 |
| CKM | 1.29 | 7.05 | 2.45 | 5.45E-04 | 6.73E-03 |
| CLCA2 | 38.90 | 6.34 | -2.62 | 2.01E-04 | 2.98E-03 |
| CLDN1 | 99.57 | 16.20 | -2.62 | 4.04E-09 | 4.06E-07 |
| CLDN14 | 1.25 | 9.64 | 2.95 | 3.35E-03 | 2.78E-02 |
| CLEC17A | 1.74 | 35.23 | 4.34 | 5.54E-06 | 1.60E-04 |
| CLEC4D | 4.36 | 25.56 | 2.55 | 1.22E-03 | 1.27E-02 |
| CLEC9A | 4.08 | 12.88 | 1.66 | 9.91E-04 | 1.07E-02 |
| CLIC5 | 3995.83 | 1161.56 | -1.78 | 1.15E-06 | 4.30E-05 |
| CLNK | 6.36 | 25.79 | 2.02 | 7.99E-04 | 9.10E-03 |
| CLSTN2 | 1461.79 | 439.08 | -1.74 | 7.37E-07 | 2.91E-05 |
| CLVS2 | 112.39 | 17.57 | -2.68 | 3.14E-05 | 6.69E-04 |
| CNKSR1 | 14.04 | 48.47 | 1.79 | 5.65E-05 | 1.08E-03 |
| CNR2 | 1.75 | 27.27 | 3.96 | 1.22E-04 | 2.01E-03 |
| CNTN1 | 167.88 | 49.61 | -1.76 | 8.56E-08 | 4.92E-06 |
| CNTN2 | 28.20 | 4.41 | -2.68 | 3.15E-04 | 4.30E-03 |
| CNTN3 | 26.46 | 3.14 | -3.07 | 9.28E-09 | 8.05E-07 |
| CNTNAP4 | 48.93 | 16.51 | -1.57 | 3.25E-04 | 4.40E-03 |
| COBL | 208.43 | 54.54 | -1.93 | 2.94E-13 | 1.50E-10 |
| COL19A1 | 3.81 | 32.63 | 3.10 | 2.96E-04 | 4.08E-03 |
| COL22A1 | 1169.29 | 285.48 | -2.03 | 7.86E-04 | 8.99E-03 |
| COL25A1 | 88.26 | 28.95 | -1.61 | 1.46E-04 | 2.32E-03 |
| COL28A1 | 86.73 | 21.78 | -1.99 | 7.47E-09 | 6.74E-07 |
| COL4A6 | 66.66 | 12.33 | -2.43 | 1.13E-05 | 2.89E-04 |
| COLGALT2 | 583.33 | 203.65 | -1.52 | 8.83E-04 | 9.87E-03 |
| CPNE5 | 91.30 | 477.54 | 2.39 | 3.88E-04 | 5.10E-03 |
| CR2 | 1.90 | 115.65 | 5.93 | 8.96E-04 | 9.96E-03 |
| CRB3 | 0.83 | 5.74 | 2.79 | 2.38E-03 | 2.14E-02 |
| CRISPLD1 | 2530.97 | 825.29 | -1.62 | 1.62E-07 | 8.53E-06 |
| CRLF1 | 7725.07 | 2283.81 | -1.76 | 9.61E-05 | 1.68E-03 |
| CRTAM | 26.16 | 90.92 | 1.80 | 1.41E-10 | 2.30E-08 |
| CRYBG2 | 2.11 | 7.84 | 1.90 | 3.43E-03 | 2.84E-02 |
| CST1 | 4.64 | 143.74 | 4.95 | 4.47E-07 | 1.95E-05 |
| CST7 | 61.66 | 246.77 | 2.00 | 3.08E-10 | 4.47E-08 |
| CTLA4 | 8.89 | 140.69 | 3.98 | 4.23E-10 | 5.70E-08 |
| CTNNA3 | 22.22 | 4.56 | -2.28 | 1.09E-04 | 1.85E-03 |
| CTSW | 53.21 | 204.20 | 1.94 | 3.90E-08 | 2.57E-06 |
| CTXND1 | 7.91 | 0.54 | -3.86 | 3.57E-06 | 1.12E-04 |
| CXCL10 | 56.74 | 599.22 | 3.40 | 9.14E-09 | 7.98E-07 |
| CXCL11 | 10.92 | 88.53 | 3.02 | 1.38E-10 | 2.27E-08 |
| CXCL13 | 6.55 | 1202.88 | 7.52 | 7.83E-08 | 4.56E-06 |
| CXCL3 | 32.45 | 151.69 | 2.22 | 2.05E-05 | 4.74E-04 |
| CXCL5 | 3.35 | 54.10 | 4.01 | 6.70E-05 | 1.24E-03 |
| CXCL9 | 137.86 | 2202.23 | 4.00 | 1.02E-12 | 3.89E-10 |
| CXCR3 | 20.85 | 127.90 | 2.62 | 6.15E-16 | 6.83E-13 |
| CXCR4 | 965.34 | 3547.62 | 1.88 | 1.04E-06 | 3.97E-05 |
| CXCR5 | 3.05 | 68.30 | 4.48 | 3.02E-06 | 9.66E-05 |
| CXCR6 | 12.63 | 74.57 | 2.56 | 9.95E-13 | 3.89E-10 |
| CXorf65 | 3.79 | 20.72 | 2.45 | 1.86E-08 | 1.41E-06 |
| CYP27B1 | 6.59 | 18.70 | 1.51 | 2.86E-04 | 3.97E-03 |
| CYTIP | 332.48 | 1200.77 | 1.85 | 1.23E-09 | 1.43E-07 |
| DAPP1 | 104.59 | 334.38 | 1.68 | 2.62E-08 | 1.89E-06 |
| DAZL | 3.77 | 57.80 | 3.94 | 2.34E-06 | 7.83E-05 |
| DBX2 | 29.47 | 6.59 | -2.16 | 2.03E-07 | 1.03E-05 |
| DCANP1 | 7.64 | 67.11 | 3.14 | 9.92E-16 | 1.04E-12 |
| DCHS2 | 16.11 | 4.88 | -1.72 | 1.65E-04 | 2.54E-03 |
| DCX | 12.74 | 1.73 | -2.88 | 8.94E-06 | 2.40E-04 |
| DEF6 | 181.96 | 605.63 | 1.73 | 1.34E-10 | 2.21E-08 |
| DENND1C | 153.53 | 444.49 | 1.53 | 2.81E-08 | 1.98E-06 |
| DERL3 | 45.63 | 1521.29 | 5.06 | 1.69E-06 | 5.97E-05 |
| DES | 936.41 | 221.34 | -2.08 | 7.15E-06 | 2.00E-04 |
| DGAT2 | 241.69 | 50.05 | -2.27 | 3.89E-03 | 3.12E-02 |
| DGKB | 15.62 | 2.28 | -2.77 | 1.67E-07 | 8.72E-06 |
| DHRS9 | 96.55 | 416.08 | 2.11 | 5.29E-12 | 1.47E-09 |
| DIO2 | 1773.22 | 616.69 | -1.52 | 1.74E-06 | 6.09E-05 |
| DIO3 | 144.32 | 11.72 | -3.62 | 3.53E-10 | 5.01E-08 |
| DKK4 | 3.87 | 0.45 | -3.10 | 4.36E-03 | 3.39E-02 |
| DNAH8 | 1.80 | 13.55 | 2.91 | 7.19E-03 | 4.95E-02 |
| DNAJC5B | 3.13 | 37.09 | 3.57 | 4.00E-07 | 1.78E-05 |
| DOK7 | 0.78 | 8.43 | 3.43 | 1.45E-04 | 2.30E-03 |
| DPP6 | 17.79 | 2.46 | -2.85 | 3.24E-04 | 4.40E-03 |
| DQX1 | 1.16 | 7.72 | 2.73 | 2.42E-04 | 3.47E-03 |
| DSCAML1 | 37.46 | 9.59 | -1.97 | 2.00E-05 | 4.64E-04 |
| DTHD1 | 13.26 | 56.15 | 2.08 | 2.10E-04 | 3.09E-03 |
| DUSP2 | 38.21 | 195.81 | 2.36 | 3.01E-05 | 6.51E-04 |
| DUSP26 | 2.08 | 10.67 | 2.36 | 2.01E-03 | 1.88E-02 |
| DUSP27 | 44.94 | 2.49 | -4.18 | 3.61E-23 | 3.41E-19 |
| EAF2 | 55.55 | 190.37 | 1.78 | 1.91E-05 | 4.46E-04 |
| ECT2L | 9.14 | 3.01 | -1.60 | 6.56E-03 | 4.63E-02 |
| EDA | 194.75 | 65.41 | -1.57 | 1.28E-12 | 4.66E-10 |
| EDN3 | 4.14 | 0.65 | -2.67 | 5.89E-03 | 4.26E-02 |
| ELANE | 73.95 | 25.84 | -1.52 | 4.17E-08 | 2.71E-06 |
| EMILIN3 | 140.74 | 28.15 | -2.32 | 1.90E-05 | 4.46E-04 |
| ENHO | 94.35 | 18.13 | -2.38 | 4.20E-03 | 3.31E-02 |
| ENPP5 | 161.32 | 44.14 | -1.87 | 7.61E-14 | 4.49E-11 |
| ENTPD3 | 82.77 | 22.49 | -1.88 | 4.44E-05 | 8.89E-04 |
| EOMES | 28.24 | 132.57 | 2.23 | 4.81E-08 | 3.04E-06 |
| EPHA1 | 8.13 | 34.65 | 2.09 | 9.75E-09 | 8.40E-07 |
| EPHA5 | 32.46 | 4.66 | -2.80 | 6.73E-08 | 4.02E-06 |
| EPHA7 | 72.45 | 14.80 | -2.29 | 1.82E-05 | 4.29E-04 |
| EPYC | 189.23 | 22.11 | -3.10 | 5.87E-04 | 7.11E-03 |
| ERBB3 | 85.42 | 24.49 | -1.80 | 2.15E-07 | 1.07E-05 |
| ERBB4 | 31.87 | 7.61 | -2.07 | 4.14E-03 | 3.28E-02 |
| ERICH3 | 102.27 | 8.74 | -3.55 | 1.42E-09 | 1.61E-07 |
| ESYT3 | 24.20 | 8.45 | -1.52 | 5.84E-05 | 1.11E-03 |
| ETV7 | 30.19 | 99.40 | 1.72 | 8.08E-13 | 3.39E-10 |
| EVPL | 43.71 | 12.47 | -1.81 | 4.90E-03 | 3.70E-02 |
| EYA1 | 32.68 | 9.28 | -1.82 | 4.11E-03 | 3.26E-02 |
| F10 | 50.84 | 8.71 | -2.55 | 1.67E-05 | 4.01E-04 |
| F5 | 2067.48 | 588.05 | -1.81 | 2.38E-04 | 3.43E-03 |
| FA2H | 3.02 | 11.05 | 1.87 | 1.83E-03 | 1.74E-02 |
| FAM129C | 11.73 | 84.93 | 2.86 | 1.30E-04 | 2.11E-03 |
| FAM169A | 7.05 | 20.78 | 1.56 | 4.33E-03 | 3.37E-02 |
| FAM181B | 16.73 | 3.67 | -2.19 | 3.87E-06 | 1.20E-04 |
| FAM46C | 221.96 | 2214.06 | 3.32 | 1.02E-03 | 1.09E-02 |
| FAM92B | 1.35 | 67.90 | 5.66 | 1.13E-04 | 1.89E-03 |
| FASLG | 6.48 | 38.47 | 2.57 | 7.67E-11 | 1.43E-08 |
| FBXL16 | 4.84 | 47.13 | 3.28 | 3.17E-06 | 1.00E-04 |
| FCAMR | 0.18 | 9.81 | 5.74 | 1.93E-03 | 1.82E-02 |
| FCGBP | 325.05 | 84.44 | -1.94 | 6.63E-07 | 2.67E-05 |
| FCGR1A | 144.89 | 621.43 | 2.10 | 2.40E-06 | 7.96E-05 |
| FCGR1B | 37.76 | 121.55 | 1.69 | 4.40E-05 | 8.84E-04 |
| FCMR | 41.29 | 361.86 | 3.13 | 2.89E-06 | 9.31E-05 |
| FCRL1 | 10.12 | 193.43 | 4.26 | 2.00E-04 | 2.97E-03 |
| FCRL2 | 2.52 | 91.85 | 5.19 | 5.63E-07 | 2.35E-05 |
| FCRL3 | 14.39 | 190.36 | 3.73 | 1.77E-07 | 9.13E-06 |
| FCRL4 | 0.13 | 4.59 | 5.16 | 2.75E-05 | 6.05E-04 |
| FCRL5 | 18.01 | 730.85 | 5.34 | 4.95E-07 | 2.13E-05 |
| FCRLA | 5.75 | 166.26 | 4.85 | 7.78E-06 | 2.13E-04 |
| FDCSP | 1.55 | 57.93 | 5.22 | 8.27E-04 | 9.37E-03 |
| FGF18 | 248.33 | 77.95 | -1.67 | 7.85E-05 | 1.41E-03 |
| FGF9 | 19.51 | 6.21 | -1.65 | 6.01E-03 | 4.33E-02 |
| FGFBP2 | 1904.27 | 576.93 | -1.72 | 1.35E-03 | 1.37E-02 |
| FKBP11 | 356.86 | 1331.52 | 1.90 | 1.75E-05 | 4.16E-04 |
| FLRT1 | 17.16 | 4.69 | -1.87 | 8.27E-04 | 9.37E-03 |
| FOLH1 | 8.35 | 26.99 | 1.69 | 5.27E-03 | 3.92E-02 |
| FOXD3 | 10.97 | 2.19 | -2.33 | 5.09E-04 | 6.37E-03 |
| FOXI2 | 15.76 | 5.19 | -1.60 | 8.37E-04 | 9.45E-03 |
| FOXP3 | 8.58 | 49.63 | 2.53 | 9.67E-10 | 1.13E-07 |
| FREM2 | 5.96 | 0.93 | -2.69 | 1.03E-03 | 1.10E-02 |
| FRMPD4 | 9.70 | 2.26 | -2.10 | 5.71E-04 | 6.98E-03 |
| FSD1 | 3.92 | 11.65 | 1.57 | 2.07E-03 | 1.92E-02 |
| FUT7 | 11.02 | 45.73 | 2.05 | 4.84E-08 | 3.05E-06 |
| GABRA2 | 11.64 | 1.94 | -2.59 | 6.03E-04 | 7.26E-03 |
| GABRA3 | 13.40 | 0.21 | -5.97 | 1.36E-12 | 4.83E-10 |
| GABRB2 | 296.56 | 75.47 | -1.97 | 4.06E-05 | 8.24E-04 |
| GALNT13 | 85.80 | 22.24 | -1.95 | 3.94E-05 | 8.04E-04 |
| GALR1 | 5.04 | 0.80 | -2.66 | 3.35E-03 | 2.78E-02 |
| GATA3 | 21.46 | 92.34 | 2.11 | 6.00E-07 | 2.48E-05 |
| GBP1 | 533.48 | 2126.68 | 2.00 | 2.37E-16 | 3.72E-13 |
| GBP5 | 114.12 | 918.84 | 3.01 | 4.38E-11 | 9.34E-09 |
| GCSAM | 7.59 | 57.12 | 2.91 | 1.05E-04 | 1.79E-03 |
| GCSAML | 39.34 | 11.24 | -1.81 | 4.19E-03 | 3.30E-02 |
| GDF10 | 321.25 | 83.32 | -1.95 | 4.53E-04 | 5.79E-03 |
| GDF5 | 155.10 | 41.07 | -1.92 | 8.62E-05 | 1.53E-03 |
| GFAP | 10.22 | 1.60 | -2.68 | 1.81E-03 | 1.73E-02 |
| GFI1 | 22.77 | 105.18 | 2.21 | 2.78E-05 | 6.11E-04 |
| GFRA1 | 515.08 | 102.06 | -2.34 | 4.40E-11 | 9.34E-09 |
| GFRA3 | 49.51 | 9.53 | -2.38 | 1.15E-03 | 1.21E-02 |
| GLDC | 4.72 | 17.45 | 1.89 | 2.07E-04 | 3.06E-03 |
| GLDN | 464.98 | 137.67 | -1.76 | 2.59E-03 | 2.29E-02 |
| GNAL | 651.95 | 158.73 | -2.04 | 5.46E-16 | 6.44E-13 |
| GNAO1 | 321.50 | 102.00 | -1.66 | 2.58E-11 | 5.87E-09 |
| GPC5 | 120.55 | 24.21 | -2.32 | 8.30E-09 | 7.32E-07 |
| GPD1 | 500.80 | 33.67 | -3.89 | 8.45E-04 | 9.51E-03 |
| GPM6A | 13.13 | 3.44 | -1.93 | 3.39E-03 | 2.81E-02 |
| GPR1 | 1761.86 | 612.56 | -1.52 | 1.44E-03 | 1.43E-02 |
| GPR12 | 34.95 | 3.28 | -3.42 | 1.27E-04 | 2.08E-03 |
| GPR15 | 0.72 | 10.89 | 3.92 | 6.30E-05 | 1.18E-03 |
| GPR171 | 16.67 | 89.96 | 2.43 | 2.30E-09 | 2.50E-07 |
| GPR174 | 6.49 | 55.61 | 3.10 | 1.02E-05 | 2.67E-04 |
| GPR18 | 4.02 | 32.72 | 3.02 | 5.05E-08 | 3.15E-06 |
| GPR20 | 19.80 | 6.67 | -1.57 | 1.02E-04 | 1.74E-03 |
| GPR25 | 0.33 | 4.97 | 3.90 | 9.05E-05 | 1.59E-03 |
| GPR84 | 12.10 | 35.37 | 1.55 | 3.78E-05 | 7.77E-04 |
| GRAMD2A | 40.31 | 8.37 | -2.27 | 3.66E-07 | 1.66E-05 |
| GREM2 | 33.30 | 8.41 | -1.99 | 5.57E-03 | 4.09E-02 |
| GRIA2 | 16.13 | 1.58 | -3.35 | 2.62E-04 | 3.70E-03 |
| GRIK3 | 195.42 | 32.50 | -2.59 | 1.53E-09 | 1.72E-07 |
| GRIK5 | 71.13 | 22.89 | -1.64 | 2.49E-03 | 2.22E-02 |
| GRIN3A | 56.45 | 19.29 | -1.55 | 2.58E-04 | 3.65E-03 |
| GRM8 | 1.59 | 6.37 | 2.01 | 6.76E-03 | 4.72E-02 |
| GSDMC | 4.30 | 38.90 | 3.18 | 2.86E-05 | 6.25E-04 |
| GSTM5 | 206.01 | 53.89 | -1.93 | 2.01E-04 | 2.98E-03 |
| GUCY2D | 5.64 | 19.81 | 1.81 | 1.82E-05 | 4.30E-04 |
| GYG2 | 114.95 | 26.15 | -2.14 | 6.53E-03 | 4.61E-02 |
| GZMA | 61.58 | 319.25 | 2.37 | 1.03E-13 | 5.88E-11 |
| GZMB | 32.92 | 190.63 | 2.53 | 8.94E-05 | 1.58E-03 |
| GZMH | 45.85 | 173.53 | 1.92 | 1.39E-08 | 1.12E-06 |
| GZMK | 66.52 | 441.11 | 2.73 | 1.60E-14 | 1.16E-11 |
| GZMM | 17.07 | 97.64 | 2.52 | 6.64E-07 | 2.67E-05 |
| HAMP | 0.90 | 12.29 | 3.77 | 5.26E-07 | 2.24E-05 |
| HCAR3 | 2.58 | 16.09 | 2.64 | 2.84E-03 | 2.46E-02 |
| HCST | 94.90 | 297.26 | 1.65 | 7.34E-17 | 1.42E-13 |
| HEPACAM | 22.72 | 1.69 | -3.75 | 7.57E-05 | 1.37E-03 |
| HHIP | 355.09 | 17.83 | -4.32 | 1.48E-07 | 7.87E-06 |
| HIST1H1C | 68.00 | 258.25 | 1.93 | 3.58E-05 | 7.43E-04 |
| HIST1H1D | 0.20 | 30.35 | 7.24 | 2.61E-03 | 2.30E-02 |
| HIST1H2BD | 17.68 | 79.18 | 2.16 | 1.16E-04 | 1.93E-03 |
| HIST1H2BG | 0.65 | 17.75 | 4.76 | 1.63E-04 | 2.51E-03 |
| HIST1H3D | 0.33 | 5.20 | 3.98 | 4.84E-04 | 6.11E-03 |
| HLA-B | 11329.54 | 35134.52 | 1.63 | 2.13E-17 | 5.26E-14 |
| HLA-DOB | 33.92 | 350.05 | 3.37 | 2.64E-04 | 3.72E-03 |
| HLA-DQA1 | 1061.28 | 5696.93 | 2.42 | 2.08E-05 | 4.81E-04 |
| HLA-DQA2 | 297.28 | 1543.58 | 2.38 | 2.06E-03 | 1.92E-02 |
| HLA-DQB1 | 1325.06 | 4925.47 | 1.89 | 1.40E-03 | 1.41E-02 |
| HLA-DRB1 | 3858.51 | 11503.94 | 1.58 | 2.31E-03 | 2.10E-02 |
| HMSD | 0.81 | 5.44 | 2.75 | 1.61E-03 | 1.57E-02 |
| HOGA1 | 87.70 | 26.08 | -1.75 | 2.75E-11 | 6.19E-09 |
| HOOK1 | 7.80 | 60.67 | 2.96 | 5.62E-03 | 4.13E-02 |
| HP | 257.72 | 20.15 | -3.68 | 1.41E-08 | 1.12E-06 |
| HPCAL4 | 2.69 | 13.45 | 2.32 | 1.47E-03 | 1.46E-02 |
| HPD | 88.35 | 9.75 | -3.18 | 2.82E-08 | 1.98E-06 |
| HPGDS | 370.10 | 124.98 | -1.57 | 3.19E-14 | 2.15E-11 |
| HPSE2 | 46.26 | 9.77 | -2.24 | 3.09E-03 | 2.62E-02 |
| HRASLS5 | 84.98 | 26.96 | -1.66 | 1.58E-08 | 1.24E-06 |
| HS3ST3B1 | 23.95 | 96.05 | 2.00 | 2.04E-07 | 1.03E-05 |
| HS6ST3 | 15.81 | 1.69 | -3.22 | 1.63E-04 | 2.52E-03 |
| HSF5 | 0.75 | 7.05 | 3.23 | 1.36E-04 | 2.19E-03 |
| HSH2D | 35.37 | 423.19 | 3.58 | 1.00E-05 | 2.63E-04 |
| HSPB7 | 235.71 | 64.10 | -1.88 | 8.99E-04 | 9.99E-03 |
| ICAM3 | 163.76 | 729.47 | 2.16 | 1.68E-05 | 4.01E-04 |
| ICAM4 | 7.92 | 30.71 | 1.96 | 3.14E-03 | 2.64E-02 |
| ICOS | 7.65 | 63.84 | 3.06 | 6.18E-08 | 3.73E-06 |
| IDO1 | 8.11 | 100.31 | 3.63 | 3.81E-13 | 1.84E-10 |
| IFNG | 2.00 | 22.12 | 3.46 | 4.80E-09 | 4.60E-07 |
| IFNLR1 | 14.73 | 64.86 | 2.14 | 1.49E-03 | 1.48E-02 |
| IGLL5 | 517.53 | 46048.66 | 6.48 | 1.84E-07 | 9.41E-06 |
| IGSF1 | 17.53 | 1.23 | -3.84 | 5.19E-12 | 1.47E-09 |
| IGSF10 | 1199.77 | 179.90 | -2.74 | 1.94E-06 | 6.62E-05 |
| IKZF3 | 98.05 | 788.82 | 3.01 | 2.67E-07 | 1.27E-05 |
| IL11 | 2.92 | 11.48 | 1.98 | 3.80E-03 | 3.08E-02 |
| IL17REL | 1.07 | 10.99 | 3.36 | 2.93E-06 | 9.39E-05 |
| IL21 | 0.12 | 4.47 | 5.20 | 1.76E-03 | 1.69E-02 |
| IL21R | 39.10 | 414.24 | 3.41 | 6.21E-08 | 3.73E-06 |
| IL23A | 3.63 | 10.48 | 1.53 | 4.95E-03 | 3.74E-02 |
| IL2RB | 98.22 | 478.98 | 2.29 | 5.64E-08 | 3.49E-06 |
| IL2RG | 144.67 | 1236.88 | 3.10 | 2.23E-17 | 5.26E-14 |
| IL32 | 408.08 | 1658.18 | 2.02 | 3.71E-21 | 2.33E-17 |
| IL4I1 | 57.56 | 543.46 | 3.24 | 9.65E-04 | 1.05E-02 |
| IL7R | 205.97 | 1633.63 | 2.99 | 6.98E-07 | 2.78E-05 |
| ILDR1 | 0.56 | 7.26 | 3.69 | 1.90E-04 | 2.85E-03 |
| INHBE | 0.13 | 3.76 | 4.90 | 1.41E-03 | 1.42E-02 |
| IPCEF1 | 30.33 | 101.18 | 1.74 | 1.19E-07 | 6.59E-06 |
| IRF1 | 454.23 | 1482.38 | 1.71 | 2.00E-08 | 1.50E-06 |
| IRF4 | 68.85 | 775.39 | 3.49 | 3.51E-05 | 7.34E-04 |
| IRX3 | 636.42 | 174.75 | -1.86 | 9.41E-08 | 5.36E-06 |
| IRX6 | 53.63 | 14.16 | -1.92 | 2.59E-05 | 5.80E-04 |
| ISG20 | 99.60 | 484.94 | 2.28 | 1.48E-04 | 2.34E-03 |
| ISL2 | 1.22 | 12.53 | 3.36 | 4.94E-04 | 6.21E-03 |
| ITGAL | 201.08 | 1024.91 | 2.35 | 5.80E-14 | 3.65E-11 |
| ITGAX | 323.17 | 1163.51 | 1.85 | 3.78E-07 | 1.70E-05 |
| ITGB7 | 96.21 | 474.13 | 2.30 | 1.05E-12 | 3.89E-10 |
| ITK | 68.75 | 357.34 | 2.38 | 1.72E-08 | 1.32E-06 |
| JAK3 | 328.24 | 1038.60 | 1.66 | 2.21E-08 | 1.65E-06 |
| JAKMIP1 | 7.78 | 46.89 | 2.59 | 4.43E-16 | 5.97E-13 |
| JCHAIN | 347.73 | 9237.36 | 4.73 | 1.25E-15 | 1.12E-12 |
| JSRP1 | 5.22 | 372.25 | 6.16 | 3.58E-07 | 1.63E-05 |
| KBTBD12 | 26.65 | 4.07 | -2.71 | 6.12E-11 | 1.22E-08 |
| KBTBD8 | 30.72 | 94.03 | 1.61 | 2.92E-04 | 4.04E-03 |
| KCNA3 | 20.54 | 156.17 | 2.93 | 2.87E-03 | 2.47E-02 |
| KCNA6 | 263.01 | 57.05 | -2.20 | 4.05E-05 | 8.21E-04 |
| KCNH1 | 92.47 | 31.26 | -1.56 | 2.59E-06 | 8.48E-05 |
| KCNIP3 | 356.81 | 101.51 | -1.81 | 1.26E-08 | 1.03E-06 |
| KCNJ10 | 2.24 | 8.30 | 1.89 | 2.30E-03 | 2.09E-02 |
| KCNJ3 | 13.01 | 2.78 | -2.23 | 5.38E-03 | 3.99E-02 |
| KCNK3 | 178.35 | 50.65 | -1.82 | 5.86E-03 | 4.25E-02 |
| KCTD16 | 24.54 | 5.18 | -2.25 | 1.47E-04 | 2.33E-03 |
| KEL | 2.79 | 11.85 | 2.09 | 1.12E-04 | 1.88E-03 |
| KIAA1324 | 13.24 | 53.64 | 2.02 | 5.40E-10 | 6.99E-08 |
| KIF26B | 17.69 | 51.00 | 1.53 | 1.64E-03 | 1.60E-02 |
| KIR2DL4 | 0.71 | 4.86 | 2.77 | 2.15E-03 | 1.99E-02 |
| KIR3DL2 | 2.08 | 8.78 | 2.08 | 8.85E-04 | 9.87E-03 |
| KIRREL2 | 0.38 | 3.36 | 3.13 | 6.91E-03 | 4.80E-02 |
| KISS1R | 0.06 | 6.04 | 6.58 | 7.30E-06 | 2.02E-04 |
| KLB | 52.17 | 4.15 | -3.65 | 1.26E-04 | 2.06E-03 |
| KLHDC7B | 6.85 | 73.53 | 3.42 | 1.59E-14 | 1.16E-11 |
| KLHL32 | 17.31 | 5.46 | -1.67 | 2.19E-03 | 2.02E-02 |
| KLHL6 | 178.97 | 847.73 | 2.24 | 3.12E-04 | 4.28E-03 |
| KLK1 | 1.42 | 16.32 | 3.52 | 3.75E-04 | 4.97E-03 |
| KLRB1 | 40.96 | 136.62 | 1.74 | 2.49E-06 | 8.18E-05 |
| KMO | 19.49 | 86.30 | 2.15 | 6.72E-12 | 1.81E-09 |
| KRT7 | 61.63 | 15.35 | -2.01 | 1.83E-03 | 1.74E-02 |
| LAD1 | 5.74 | 30.70 | 2.42 | 1.42E-05 | 3.51E-04 |
| LAIR2 | 1.85 | 22.51 | 3.61 | 4.24E-12 | 1.29E-09 |
| LAMA2 | 2330.96 | 663.06 | -1.81 | 9.06E-04 | 1.01E-02 |
| LAMP3 | 15.80 | 250.08 | 3.98 | 2.61E-07 | 1.25E-05 |
| LAMP5 | 7.41 | 52.60 | 2.83 | 7.64E-07 | 3.01E-05 |
| LARGE2 | 2.86 | 19.41 | 2.76 | 1.78E-06 | 6.20E-05 |
| LAX1 | 58.34 | 592.49 | 3.34 | 1.21E-04 | 1.99E-03 |
| LCK | 65.11 | 464.72 | 2.84 | 9.38E-11 | 1.65E-08 |
| LEF1 | 106.46 | 333.46 | 1.65 | 2.73E-06 | 8.88E-05 |
| LGALS12 | 39.42 | 8.00 | -2.30 | 1.12E-04 | 1.87E-03 |
| LGALS2 | 29.85 | 111.12 | 1.90 | 1.22E-15 | 1.12E-12 |
| LGI1 | 29.99 | 9.12 | -1.72 | 1.10E-03 | 1.16E-02 |
| LHCGR | 29.59 | 6.98 | -2.08 | 1.14E-03 | 1.20E-02 |
| LHX9 | 91.03 | 30.47 | -1.58 | 4.23E-04 | 5.49E-03 |
| LIMD2 | 223.77 | 946.52 | 2.08 | 4.88E-12 | 1.42E-09 |
| LIME1 | 94.84 | 391.90 | 2.05 | 2.67E-06 | 8.69E-05 |
| LIPE | 270.92 | 65.83 | -2.04 | 4.19E-03 | 3.30E-02 |
| LMO3 | 194.09 | 34.69 | -2.48 | 1.40E-03 | 1.41E-02 |
| LOC100509620 | 11.16 | 0.51 | -4.46 | 7.95E-05 | 1.43E-03 |
| LOC101927401 | 11.95 | 2.72 | -2.13 | 3.99E-04 | 5.22E-03 |
| LOC102724971 | 0.14 | 12.51 | 6.50 | 5.65E-03 | 4.14E-02 |
| LOC105378577 | 4.08 | 0.23 | -4.13 | 5.87E-04 | 7.11E-03 |
| LOC107984851 | 18.33 | 5.34 | -1.78 | 1.59E-03 | 1.56E-02 |
| LOC107985532 | 3.08 | 13.34 | 2.12 | 1.11E-03 | 1.17E-02 |
| LOC107985795 | 5.39 | 0.21 | -4.68 | 1.02E-04 | 1.75E-03 |
| LOC107987211 | 0.06 | 4.34 | 6.07 | 2.75E-03 | 2.40E-02 |
| LOC112267934 | 4.81 | 0.95 | -2.34 | 6.97E-03 | 4.83E-02 |
| LOC283710 | 2.31 | 180.94 | 6.29 | 7.31E-09 | 6.63E-07 |
| LOC390937 | 3.89 | 17.63 | 2.18 | 3.16E-03 | 2.66E-02 |
| LOC401478 | 9.26 | 2.39 | -1.96 | 1.91E-03 | 1.80E-02 |
| LOC644090 | 1.15 | 8.63 | 2.91 | 4.35E-04 | 5.62E-03 |
| LONRF2 | 104.50 | 33.66 | -1.63 | 1.48E-06 | 5.32E-05 |
| LPL | 958.14 | 279.98 | -1.77 | 7.13E-03 | 4.92E-02 |
| LRFN5 | 122.93 | 24.12 | -2.35 | 1.26E-05 | 3.18E-04 |
| LRMP | 141.54 | 406.93 | 1.52 | 2.89E-07 | 1.36E-05 |
| LRRC26 | 0.38 | 4.11 | 3.43 | 1.27E-03 | 1.30E-02 |
| LRRTM4 | 3.54 | 0.13 | -4.80 | 5.96E-04 | 7.20E-03 |
| LTA | 5.26 | 30.59 | 2.54 | 4.64E-07 | 2.01E-05 |
| LTB | 57.83 | 614.23 | 3.41 | 2.07E-06 | 7.00E-05 |
| LTBP4 | 14203.23 | 4441.58 | -1.68 | 2.48E-10 | 3.71E-08 |
| LY6H | 5.68 | 21.43 | 1.92 | 1.40E-03 | 1.41E-02 |
| LY9 | 17.30 | 165.85 | 3.26 | 6.36E-05 | 1.19E-03 |
| LYPD6 | 9.72 | 2.78 | -1.80 | 1.91E-03 | 1.80E-02 |
| LYPD6B | 0.45 | 6.36 | 3.84 | 6.44E-03 | 4.57E-02 |
| LYVE1 | 5003.54 | 1588.13 | -1.66 | 1.67E-03 | 1.62E-02 |
| LYZ | 5136.13 | 16467.13 | 1.68 | 6.17E-07 | 2.53E-05 |
| MAB21L1 | 83.77 | 28.71 | -1.54 | 6.58E-09 | 6.01E-07 |
| MAP4K1 | 115.91 | 428.01 | 1.88 | 5.37E-07 | 2.27E-05 |
| MAPK4 | 21.87 | 2.73 | -3.00 | 3.25E-04 | 4.41E-03 |
| MARC1 | 124.85 | 43.68 | -1.52 | 6.26E-03 | 4.46E-02 |
| MARCKSL1 | 423.89 | 1329.87 | 1.65 | 3.40E-04 | 4.58E-03 |
| MASP1 | 168.55 | 43.35 | -1.96 | 1.84E-05 | 4.33E-04 |
| MAT1A | 2.12 | 23.07 | 3.44 | 3.02E-03 | 2.57E-02 |
| MC4R | 0.45 | 20.64 | 5.52 | 1.56E-03 | 1.53E-02 |
| MDFIC2 | 3.30 | 0.23 | -3.84 | 5.44E-03 | 4.03E-02 |
| MEI1 | 26.26 | 290.90 | 3.47 | 1.85E-03 | 1.76E-02 |
| METTL24 | 30.26 | 6.46 | -2.23 | 4.66E-09 | 4.49E-07 |
| MGAT4C | 113.53 | 40.11 | -1.50 | 4.07E-03 | 3.23E-02 |
| MIXL1 | 1.29 | 44.03 | 5.09 | 3.23E-05 | 6.87E-04 |
| MMP10 | 13.09 | 86.33 | 2.72 | 2.47E-03 | 2.21E-02 |
| MMP12 | 3.26 | 62.18 | 4.25 | 1.01E-03 | 1.09E-02 |
| MMP3 | 16373.28 | 114559.38 | 2.81 | 5.26E-03 | 3.92E-02 |
| MOXD1 | 30.14 | 137.01 | 2.18 | 3.08E-05 | 6.61E-04 |
| MPIG6B | 11.71 | 3.89 | -1.59 | 3.01E-03 | 2.56E-02 |
| MPP4 | 2.69 | 8.17 | 1.60 | 6.15E-03 | 4.40E-02 |
| MPPED2 | 125.07 | 40.53 | -1.63 | 2.84E-07 | 1.34E-05 |
| MPV17L | 61.09 | 20.04 | -1.61 | 1.12E-08 | 9.36E-07 |
| MRAP | 10.73 | 1.69 | -2.67 | 2.26E-04 | 3.29E-03 |
| MRGPRE | 11.66 | 1.36 | -3.10 | 6.55E-07 | 2.65E-05 |
| MRGPRX2 | 6.32 | 0.63 | -3.32 | 2.78E-03 | 2.42E-02 |
| MS4A1 | 33.43 | 1044.82 | 4.97 | 1.11E-06 | 4.19E-05 |
| MT1A | 92.48 | 25.65 | -1.85 | 3.93E-03 | 3.13E-02 |
| MUC16 | 1.19 | 6.60 | 2.47 | 3.00E-03 | 2.56E-02 |
| MYBL2 | 50.24 | 163.47 | 1.70 | 1.98E-05 | 4.61E-04 |
| MYBPC1 | 1.00 | 6.24 | 2.64 | 7.28E-04 | 8.44E-03 |
| MYBPC2 | 0.57 | 10.26 | 4.17 | 1.33E-03 | 1.36E-02 |
| MYH1 | 1.63 | 6.87 | 2.07 | 3.10E-03 | 2.62E-02 |
| MYH14 | 55.16 | 12.96 | -2.09 | 2.38E-04 | 3.43E-03 |
| MYH2 | 1.28 | 10.26 | 3.01 | 2.54E-05 | 5.70E-04 |
| MYH7 | 0.38 | 5.21 | 3.79 | 1.18E-04 | 1.96E-03 |
| MYL3 | 27.40 | 8.98 | -1.61 | 4.62E-04 | 5.87E-03 |
| MYO3A | 7.74 | 1.77 | -2.13 | 4.26E-03 | 3.34E-02 |
| MYOCD | 164.60 | 43.73 | -1.91 | 1.59E-10 | 2.57E-08 |
| MYOT | 18.88 | 3.04 | -2.64 | 7.14E-07 | 2.84E-05 |
| MYPN | 263.40 | 74.55 | -1.82 | 1.66E-05 | 3.99E-04 |
| MZB1 | 35.22 | 2463.13 | 6.13 | 2.87E-08 | 2.01E-06 |
| NAALAD2 | 86.85 | 22.21 | -1.97 | 6.38E-08 | 3.82E-06 |
| NAT8L | 49.70 | 5.39 | -3.20 | 2.81E-03 | 2.43E-02 |
| NBPF4 | 1.41 | 5.97 | 2.08 | 7.00E-03 | 4.85E-02 |
| NCR3 | 5.64 | 18.54 | 1.72 | 6.39E-05 | 1.20E-03 |
| NDNF | 3112.98 | 577.03 | -2.43 | 4.33E-11 | 9.34E-09 |
| NDST4 | 3.30 | 0.14 | -4.59 | 1.07E-03 | 1.14E-02 |
| NEGR1 | 455.85 | 112.08 | -2.02 | 1.05E-08 | 8.83E-07 |
| NELL1 | 174.32 | 29.65 | -2.56 | 3.51E-03 | 2.89E-02 |
| NELL2 | 27.46 | 84.84 | 1.63 | 5.83E-03 | 4.23E-02 |
| NETO2 | 34.33 | 119.13 | 1.79 | 4.54E-08 | 2.91E-06 |
| NFKBIE | 127.54 | 367.31 | 1.53 | 2.56E-11 | 5.87E-09 |
| NKAIN2 | 15.36 | 3.95 | -1.96 | 6.40E-05 | 1.20E-03 |
| NKD1 | 235.45 | 66.86 | -1.82 | 5.01E-11 | 1.03E-08 |
| NKG7 | 81.89 | 341.44 | 2.06 | 1.41E-09 | 1.61E-07 |
| NLGN1 | 67.60 | 10.70 | -2.66 | 3.45E-18 | 1.12E-14 |
| NLRC3 | 62.10 | 335.31 | 2.43 | 3.20E-09 | 3.30E-07 |
| NLRP2 | 7.79 | 50.70 | 2.70 | 5.27E-06 | 1.54E-04 |
| NLRP7 | 0.19 | 7.42 | 5.28 | 1.13E-05 | 2.90E-04 |
| NME8 | 7.57 | 28.61 | 1.92 | 3.00E-05 | 6.51E-04 |
| NNAT | 90.94 | 23.59 | -1.95 | 1.11E-04 | 1.87E-03 |
| NOS1AP | 24.40 | 5.17 | -2.24 | 3.11E-08 | 2.16E-06 |
| NOX5 | 14.25 | 3.80 | -1.91 | 4.57E-03 | 3.51E-02 |
| NPC1L1 | 10.93 | 2.45 | -2.15 | 1.56E-04 | 2.44E-03 |
| NPR3 | 631.05 | 142.36 | -2.15 | 3.03E-06 | 9.69E-05 |
| NPY5R | 9.61 | 1.69 | -2.51 | 9.66E-05 | 1.68E-03 |
| NRXN1 | 70.67 | 19.38 | -1.87 | 1.09E-04 | 1.84E-03 |
| NT5DC4 | 0.78 | 6.66 | 3.09 | 7.53E-05 | 1.36E-03 |
| NTN1 | 1811.15 | 573.79 | -1.66 | 6.36E-06 | 1.80E-04 |
| NTRK1 | 46.58 | 15.32 | -1.60 | 1.49E-07 | 7.92E-06 |
| NTRK2 | 3146.76 | 801.68 | -1.97 | 1.08E-07 | 5.99E-06 |
| NTRK3 | 178.86 | 61.00 | -1.55 | 6.53E-05 | 1.22E-03 |
| NTSR1 | 7.15 | 22.83 | 1.68 | 3.30E-04 | 4.46E-03 |
| NUAK2 | 19.78 | 99.68 | 2.33 | 4.27E-10 | 5.72E-08 |
| NUGGC | 6.62 | 126.36 | 4.25 | 6.14E-05 | 1.16E-03 |
| NUP210 | 163.74 | 752.08 | 2.20 | 7.25E-05 | 1.32E-03 |
| NXPE2 | 34.50 | 9.43 | -1.87 | 1.81E-05 | 4.28E-04 |
| OR2W3 | 8.55 | 2.81 | -1.61 | 6.59E-03 | 4.64E-02 |
| OTOF | 65.00 | 11.28 | -2.53 | 9.82E-04 | 1.06E-02 |
| P2RX5 | 29.14 | 176.96 | 2.60 | 1.73E-03 | 1.67E-02 |
| P2RY10 | 17.78 | 204.49 | 3.52 | 2.98E-08 | 2.08E-06 |
| P2RY8 | 1.47 | 6.57 | 2.16 | 3.13E-03 | 2.64E-02 |
| PACSIN1 | 6.29 | 24.07 | 1.94 | 7.18E-06 | 2.00E-04 |
| PALM3 | 16.77 | 5.91 | -1.51 | 5.79E-04 | 7.03E-03 |
| PAPPA2 | 34.41 | 5.12 | -2.75 | 3.12E-06 | 9.92E-05 |
| PAX1 | 16.27 | 2.02 | -3.01 | 9.92E-04 | 1.07E-02 |
| PAX5 | 6.66 | 142.76 | 4.42 | 3.22E-04 | 4.37E-03 |
| PCDH17 | 141.98 | 416.27 | 1.55 | 3.99E-05 | 8.14E-04 |
| PCDH20 | 14.23 | 3.57 | -1.99 | 2.48E-03 | 2.22E-02 |
| PCDH8 | 7.34 | 0.62 | -3.56 | 1.27E-03 | 1.31E-02 |
| PCDHA10 | 8.96 | 2.96 | -1.60 | 5.38E-03 | 3.99E-02 |
| PCDHA12 | 4.34 | 0.11 | -5.34 | 1.01E-04 | 1.74E-03 |
| PCDHA13 | 13.32 | 2.24 | -2.57 | 2.38E-05 | 5.39E-04 |
| PCDHA3 | 21.24 | 6.72 | -1.66 | 4.31E-05 | 8.67E-04 |
| PCDHA6 | 11.54 | 3.32 | -1.80 | 5.47E-04 | 6.75E-03 |
| PCDHAC2 | 33.14 | 7.65 | -2.11 | 2.88E-06 | 9.26E-05 |
| PCDHB16 | 282.63 | 97.58 | -1.53 | 6.37E-09 | 5.89E-07 |
| PCDHB6 | 69.96 | 24.39 | -1.52 | 7.21E-08 | 4.27E-06 |
| PCDHGA2 | 102.87 | 23.29 | -2.14 | 2.89E-13 | 1.50E-10 |
| PCDHGA3 | 63.92 | 18.60 | -1.78 | 2.87E-07 | 1.35E-05 |
| PCDHGA4 | 58.51 | 20.58 | -1.51 | 9.15E-07 | 3.54E-05 |
| PCDHGA5 | 101.64 | 27.82 | -1.87 | 5.53E-09 | 5.21E-07 |
| PCDHGA7 | 38.93 | 13.54 | -1.52 | 1.57E-06 | 5.60E-05 |
| PCDHGB1 | 23.11 | 7.90 | -1.55 | 6.65E-05 | 1.23E-03 |
| PCDHGB2 | 102.00 | 33.21 | -1.62 | 9.95E-09 | 8.54E-07 |
| PCK1 | 253.92 | 21.53 | -3.56 | 1.34E-08 | 1.08E-06 |
| PCOLCE2 | 5359.34 | 1735.45 | -1.63 | 1.93E-06 | 6.62E-05 |
| PCP2 | 6.61 | 24.68 | 1.90 | 1.74E-04 | 2.65E-03 |
| PCP4L1 | 20.33 | 6.96 | -1.55 | 2.72E-03 | 2.38E-02 |
| PCSK2 | 47.80 | 8.59 | -2.48 | 2.93E-03 | 2.52E-02 |
| PDCD1 | 12.52 | 159.29 | 3.67 | 4.60E-19 | 2.17E-15 |
| PDE11A | 13.19 | 2.28 | -2.53 | 6.52E-03 | 4.61E-02 |
| PDE6G | 4.67 | 15.35 | 1.72 | 1.85E-04 | 2.79E-03 |
| PDK1 | 198.33 | 936.18 | 2.24 | 9.39E-05 | 1.65E-03 |
| PDK4 | 9982.33 | 3199.54 | -1.64 | 8.90E-04 | 9.90E-03 |
| PDZRN4 | 149.49 | 35.33 | -2.08 | 4.54E-03 | 3.50E-02 |
| PENK | 828.46 | 31.80 | -4.70 | 1.80E-03 | 1.73E-02 |
| PGA3 | 14.72 | 3.01 | -2.29 | 6.82E-05 | 1.25E-03 |
| PGLYRP2 | 0.72 | 7.01 | 3.28 | 1.11E-04 | 1.87E-03 |
| PIM2 | 136.73 | 2591.59 | 4.24 | 4.44E-05 | 8.89E-04 |
| PKD1L2 | 155.27 | 52.96 | -1.55 | 6.05E-03 | 4.35E-02 |
| PLA2G2D | 7.71 | 465.76 | 5.92 | 7.94E-09 | 7.04E-07 |
| PLA2G7 | 22.00 | 103.03 | 2.23 | 1.53E-06 | 5.47E-05 |
| PLAC8 | 16.23 | 58.09 | 1.84 | 3.45E-05 | 7.25E-04 |
| PLCXD3 | 216.63 | 38.19 | -2.50 | 6.25E-04 | 7.48E-03 |
| PLEKHG7 | 0.51 | 8.04 | 3.99 | 1.86E-03 | 1.77E-02 |
| PLIN1 | 1068.76 | 65.80 | -4.02 | 1.39E-04 | 2.24E-03 |
| PLIN4 | 2124.26 | 126.10 | -4.07 | 1.24E-06 | 4.55E-05 |
| PLIN5 | 35.18 | 5.26 | -2.74 | 4.62E-03 | 3.54E-02 |
| PLPP3 | 7762.40 | 2173.36 | -1.84 | 4.93E-16 | 6.20E-13 |
| PMP2 | 22.29 | 6.95 | -1.68 | 5.65E-03 | 4.14E-02 |
| PNOC | 1.60 | 68.65 | 5.42 | 1.30E-07 | 7.07E-06 |
| PODXL2 | 180.29 | 63.47 | -1.51 | 1.66E-08 | 1.29E-06 |
| POU2AF1 | 35.15 | 1473.11 | 5.39 | 6.75E-07 | 2.71E-05 |
| POU3F1 | 4.01 | 15.31 | 1.93 | 7.09E-04 | 8.26E-03 |
| POU6F2 | 3.73 | 0.11 | -5.12 | 4.14E-04 | 5.37E-03 |
| PPDPFL | 12.19 | 0.78 | -3.97 | 2.37E-07 | 1.16E-05 |
| PPL | 2520.18 | 613.46 | -2.04 | 9.73E-06 | 2.57E-04 |
| PRDM1 | 241.48 | 1401.64 | 2.54 | 9.82E-04 | 1.06E-02 |
| PRELP | 22631.71 | 7920.81 | -1.51 | 1.93E-05 | 4.50E-04 |
| PRIMA1 | 50.75 | 10.83 | -2.23 | 5.14E-06 | 1.51E-04 |
| PRKAA2 | 75.13 | 25.30 | -1.57 | 4.29E-08 | 2.78E-06 |
| PRKCB | 174.93 | 529.73 | 1.60 | 3.67E-05 | 7.58E-04 |
| PRKCQ | 20.79 | 86.19 | 2.05 | 2.27E-08 | 1.68E-06 |
| PROK1 | 14.50 | 0.81 | -4.16 | 1.36E-03 | 1.38E-02 |
| PRPH | 75.90 | 20.65 | -1.88 | 1.83E-08 | 1.40E-06 |
| PRSS1 | 3.65 | 21.63 | 2.57 | 1.59E-03 | 1.56E-02 |
| PRSS16 | 1.24 | 27.77 | 4.49 | 1.37E-04 | 2.21E-03 |
| PSAT1 | 38.29 | 137.04 | 1.84 | 2.70E-03 | 2.37E-02 |
| PTCHD1 | 10.13 | 0.62 | -4.02 | 9.17E-06 | 2.46E-04 |
| PTGER1 | 47.96 | 10.17 | -2.24 | 5.80E-05 | 1.11E-03 |
| PTPN7 | 74.48 | 448.50 | 2.59 | 1.76E-12 | 5.83E-10 |
| PTPRCAP | 83.51 | 668.46 | 3.00 | 5.86E-10 | 7.48E-08 |
| PTPRF | 1963.24 | 659.86 | -1.57 | 3.85E-16 | 5.59E-13 |
| PTPRZ1 | 105.31 | 28.42 | -1.89 | 4.98E-04 | 6.25E-03 |
| PVRIG | 13.60 | 76.66 | 2.49 | 5.92E-07 | 2.46E-05 |
| PXDNL | 229.05 | 72.82 | -1.65 | 6.28E-04 | 7.50E-03 |
| PYHIN1 | 27.81 | 142.37 | 2.36 | 2.30E-07 | 1.13E-05 |
| QPCT | 68.18 | 241.31 | 1.82 | 5.76E-08 | 3.55E-06 |
| QRFPR | 4.92 | 0.56 | -3.14 | 1.15E-03 | 1.21E-02 |
| RAB11FIP4 | 44.89 | 140.65 | 1.65 | 3.80E-06 | 1.18E-04 |
| RAB26 | 7.03 | 32.15 | 2.19 | 3.38E-03 | 2.80E-02 |
| RAB39B | 8.37 | 60.29 | 2.85 | 1.04E-04 | 1.77E-03 |
| RAC2 | 299.23 | 1224.54 | 2.03 | 8.76E-09 | 7.69E-07 |
| RALGPS2 | 207.29 | 635.70 | 1.62 | 4.76E-04 | 6.03E-03 |
| RASGEF1C | 8.99 | 2.02 | -2.15 | 6.98E-04 | 8.16E-03 |
| RASGRP1 | 44.53 | 247.46 | 2.47 | 1.96E-07 | 9.95E-06 |
| RASSF10 | 9.75 | 1.90 | -2.36 | 1.12E-04 | 1.88E-03 |
| RASSF6 | 5.50 | 145.07 | 4.72 | 3.41E-03 | 2.83E-02 |
| RBP4 | 775.70 | 215.25 | -1.85 | 4.07E-05 | 8.24E-04 |
| RELN | 117.71 | 28.49 | -2.05 | 5.60E-05 | 1.08E-03 |
| RGL4 | 9.10 | 27.87 | 1.61 | 2.75E-05 | 6.05E-04 |
| RGMA | 1689.59 | 582.86 | -1.54 | 1.04E-09 | 1.21E-07 |
| RGPD6 | 28.35 | 8.89 | -1.67 | 1.17E-03 | 1.22E-02 |
| RGS1 | 1216.64 | 3552.11 | 1.55 | 4.65E-06 | 1.40E-04 |
| RGS6 | 137.63 | 48.07 | -1.52 | 8.27E-04 | 9.37E-03 |
| RHOF | 64.14 | 221.72 | 1.79 | 4.28E-07 | 1.89E-05 |
| RHOH | 30.37 | 235.71 | 2.96 | 8.36E-06 | 2.27E-04 |
| RIMS1 | 13.38 | 1.43 | -3.22 | 1.69E-03 | 1.63E-02 |
| ROBO2 | 69.71 | 19.85 | -1.81 | 2.42E-07 | 1.18E-05 |
| RRM2 | 98.54 | 281.26 | 1.51 | 4.58E-05 | 9.13E-04 |
| RSPO1 | 5.45 | 1.14 | -2.26 | 5.69E-03 | 4.15E-02 |
| RSPO4 | 41.06 | 6.63 | -2.63 | 7.46E-05 | 1.35E-03 |
| RTKN2 | 15.31 | 46.52 | 1.60 | 3.86E-07 | 1.73E-05 |
| RTP5 | 1.99 | 13.01 | 2.71 | 3.58E-05 | 7.43E-04 |
| RYR3 | 155.16 | 40.93 | -1.92 | 2.61E-05 | 5.82E-04 |
| S1PR4 | 22.35 | 114.79 | 2.36 | 2.80E-10 | 4.10E-08 |
| SAMD10 | 21.85 | 80.54 | 1.88 | 2.11E-07 | 1.06E-05 |
| SAMD3 | 31.46 | 128.20 | 2.03 | 1.30E-10 | 2.20E-08 |
| SBSN | 14.07 | 59.41 | 2.08 | 4.06E-03 | 3.23E-02 |
| SBSPON | 174.66 | 45.00 | -1.96 | 1.12E-08 | 9.36E-07 |
| SCG2 | 2156.68 | 667.86 | -1.69 | 5.20E-04 | 6.47E-03 |
| SCML4 | 37.87 | 142.69 | 1.91 | 1.50E-04 | 2.36E-03 |
| SCN4A | 65.32 | 16.48 | -1.99 | 1.83E-06 | 6.32E-05 |
| SCN7A | 60.60 | 10.22 | -2.57 | 1.50E-05 | 3.68E-04 |
| SCUBE1 | 996.38 | 290.66 | -1.78 | 9.55E-04 | 1.05E-02 |
| SCUBE3 | 86.46 | 28.89 | -1.58 | 4.97E-04 | 6.23E-03 |
| SEC11C | 339.56 | 1142.20 | 1.75 | 1.78E-04 | 2.71E-03 |
| SEC14L5 | 41.99 | 12.30 | -1.77 | 1.13E-07 | 6.28E-06 |
| SEL1L3 | 498.41 | 2174.23 | 2.13 | 1.95E-04 | 2.92E-03 |
| SEMA3E | 1043.36 | 227.89 | -2.19 | 7.33E-11 | 1.38E-08 |
| SEMA4A | 141.60 | 426.01 | 1.59 | 5.93E-08 | 3.61E-06 |
| SEMA4D | 250.15 | 830.11 | 1.73 | 1.90E-05 | 4.46E-04 |
| SEPT1 | 133.94 | 513.24 | 1.94 | 2.74E-06 | 8.88E-05 |
| SERPINA1 | 454.70 | 2553.26 | 2.49 | 2.47E-08 | 1.79E-06 |
| SERPINA3 | 1349.58 | 395.82 | -1.77 | 1.43E-06 | 5.16E-05 |
| SERPINA5 | 575.18 | 121.87 | -2.24 | 4.96E-09 | 4.73E-07 |
| SERPINA9 | 0.37 | 5.28 | 3.82 | 3.87E-03 | 3.12E-02 |
| SEZ6L2 | 78.37 | 223.94 | 1.51 | 1.90E-03 | 1.80E-02 |
| SFRP1 | 8919.41 | 1780.61 | -2.32 | 8.28E-17 | 1.42E-13 |
| SFRP5 | 14.63 | 0.91 | -4.01 | 4.93E-06 | 1.45E-04 |
| SGCG | 11.88 | 2.47 | -2.27 | 7.28E-04 | 8.44E-03 |
| SGPP2 | 6.75 | 52.93 | 2.97 | 4.57E-07 | 1.98E-05 |
| SH2D1A | 41.12 | 188.99 | 2.20 | 5.08E-10 | 6.70E-08 |
| SH2D2A | 43.19 | 126.89 | 1.55 | 6.59E-09 | 6.01E-07 |
| SH2D3A | 14.30 | 43.24 | 1.60 | 4.69E-05 | 9.30E-04 |
| SH3GL2 | 26.65 | 7.71 | -1.79 | 1.92E-04 | 2.88E-03 |
| SHANK2 | 235.20 | 65.76 | -1.84 | 2.31E-05 | 5.25E-04 |
| SHD | 0.58 | 5.65 | 3.28 | 2.00E-04 | 2.97E-03 |
| SHISA8 | 1.99 | 28.65 | 3.84 | 8.34E-04 | 9.42E-03 |
| SHISAL2A | 2.53 | 17.76 | 2.81 | 4.57E-05 | 9.12E-04 |
| SIGLEC10 | 110.92 | 350.23 | 1.66 | 8.30E-05 | 1.48E-03 |
| SIGLEC12 | 10.01 | 30.37 | 1.60 | 3.97E-04 | 5.21E-03 |
| SIRPG | 6.05 | 92.98 | 3.94 | 2.73E-10 | 4.03E-08 |
| SIT1 | 16.48 | 127.43 | 2.95 | 1.96E-06 | 6.69E-05 |
| SLA2 | 21.51 | 100.18 | 2.22 | 5.38E-13 | 2.36E-10 |
| SLAMF1 | 17.41 | 172.90 | 3.31 | 1.62E-10 | 2.60E-08 |
| SLAMF6 | 74.15 | 445.07 | 2.59 | 7.93E-09 | 7.04E-07 |
| SLAMF7 | 68.91 | 1799.75 | 4.71 | 2.43E-08 | 1.77E-06 |
| SLAMF8 | 208.74 | 1522.55 | 2.87 | 2.48E-09 | 2.64E-07 |
| SLAMF9 | 2.07 | 20.43 | 3.30 | 2.55E-03 | 2.26E-02 |
| SLC12A3 | 1.07 | 6.89 | 2.69 | 3.97E-04 | 5.21E-03 |
| SLC12A5 | 4.02 | 11.78 | 1.55 | 5.46E-03 | 4.03E-02 |
| SLC12A8 | 41.89 | 155.31 | 1.89 | 1.49E-05 | 3.64E-04 |
| SLC14A2 | 13.83 | 4.46 | -1.63 | 9.66E-04 | 1.05E-02 |
| SLC23A1 | 1.05 | 6.63 | 2.66 | 6.63E-04 | 7.84E-03 |
| SLC26A7 | 12.65 | 52.65 | 2.06 | 3.59E-03 | 2.94E-02 |
| SLC27A2 | 3.28 | 15.55 | 2.25 | 3.83E-06 | 1.19E-04 |
| SLC27A6 | 15.32 | 1.91 | -3.00 | 7.01E-05 | 1.28E-03 |
| SLC28A3 | 11.83 | 95.72 | 3.02 | 1.01E-04 | 1.74E-03 |
| SLC38A5 | 113.77 | 337.43 | 1.57 | 2.35E-06 | 7.83E-05 |
| SLC47A1 | 240.24 | 72.66 | -1.73 | 4.29E-07 | 1.89E-05 |
| SLC5A9 | 6.19 | 0.66 | -3.23 | 2.37E-04 | 3.42E-03 |
| SLC6A2 | 145.07 | 18.76 | -2.95 | 7.48E-10 | 9.28E-08 |
| SLC6A4 | 4.83 | 0.69 | -2.82 | 4.56E-03 | 3.51E-02 |
| SLC7A10 | 22.75 | 2.69 | -3.08 | 9.11E-04 | 1.01E-02 |
| SLC7A5 | 174.95 | 648.31 | 1.89 | 2.84E-04 | 3.95E-03 |
| SLFN12L | 19.53 | 56.55 | 1.53 | 7.46E-05 | 1.35E-03 |
| SLITRK6 | 40.77 | 6.34 | -2.69 | 4.46E-04 | 5.73E-03 |
| SLPI | 255.64 | 72.20 | -1.82 | 3.50E-05 | 7.33E-04 |
| SMPDL3B | 8.53 | 146.23 | 4.10 | 1.15E-03 | 1.21E-02 |
| SNX20 | 140.37 | 443.23 | 1.66 | 4.38E-09 | 4.26E-07 |
| SOCS1 | 67.93 | 227.18 | 1.74 | 2.42E-09 | 2.60E-07 |
| SORCS1 | 60.05 | 9.81 | -2.61 | 1.16E-05 | 2.94E-04 |
| SOSTDC1 | 3.42 | 0.12 | -4.84 | 2.01E-03 | 1.88E-02 |
| SOWAHD | 14.39 | 43.01 | 1.58 | 2.30E-07 | 1.13E-05 |
| SOX10 | 54.64 | 13.22 | -2.05 | 1.56E-04 | 2.43E-03 |
| SOX6 | 150.89 | 53.06 | -1.51 | 4.07E-11 | 8.93E-09 |
| SP140 | 63.67 | 311.08 | 2.29 | 9.30E-09 | 8.05E-07 |
| SPAG4 | 25.00 | 349.95 | 3.81 | 5.08E-03 | 3.81E-02 |
| SPIB | 5.22 | 162.80 | 4.96 | 6.46E-06 | 1.82E-04 |
| SPN | 90.80 | 336.59 | 1.89 | 8.16E-06 | 2.23E-04 |
| SPOCK2 | 237.32 | 777.01 | 1.71 | 2.10E-05 | 4.83E-04 |
| SPP1 | 1161.29 | 5978.03 | 2.36 | 2.10E-03 | 1.95E-02 |
| SSR4 | 1801.04 | 5760.46 | 1.68 | 1.62E-04 | 2.51E-03 |
| SSTR1 | 21.05 | 3.75 | -2.49 | 2.64E-08 | 1.89E-06 |
| SSTR3 | 0.97 | 15.21 | 3.97 | 1.44E-04 | 2.30E-03 |
| ST6GAL2 | 38.32 | 138.35 | 1.85 | 5.87E-03 | 4.25E-02 |
| STAP1 | 15.00 | 86.48 | 2.53 | 2.31E-03 | 2.10E-02 |
| STMN2 | 148.07 | 24.88 | -2.57 | 1.15E-05 | 2.93E-04 |
| SUSD3 | 27.68 | 83.85 | 1.60 | 3.10E-05 | 6.64E-04 |
| SUSD5 | 953.05 | 335.49 | -1.51 | 5.21E-10 | 6.82E-08 |
| SV2B | 876.30 | 288.24 | -1.60 | 5.58E-05 | 1.07E-03 |
| SYNM | 1549.98 | 523.81 | -1.57 | 2.12E-07 | 1.06E-05 |
| SYT1 | 122.85 | 39.72 | -1.63 | 5.66E-10 | 7.27E-08 |
| SYT13 | 19.64 | 1.72 | -3.51 | 1.20E-04 | 1.98E-03 |
| SYTL1 | 43.84 | 412.55 | 3.23 | 5.08E-05 | 9.97E-04 |
| TARP | 26.74 | 138.45 | 2.37 | 1.57E-08 | 1.24E-06 |
| TAS1R3 | 12.80 | 116.71 | 3.19 | 8.17E-04 | 9.27E-03 |
| TBC1D10C | 68.30 | 483.52 | 2.82 | 3.99E-10 | 5.55E-08 |
| TBXT | 11.47 | 0.81 | -3.82 | 8.23E-06 | 2.24E-04 |
| TCEAL2 | 81.09 | 24.29 | -1.74 | 8.52E-05 | 1.51E-03 |
| TCF23 | 6.85 | 0.46 | -3.89 | 1.06E-05 | 2.74E-04 |
| TCF7 | 73.03 | 538.37 | 2.88 | 1.13E-06 | 4.24E-05 |
| TCHH | 70.69 | 20.67 | -1.77 | 1.06E-03 | 1.13E-02 |
| TCL1A | 3.31 | 56.44 | 4.09 | 7.49E-04 | 8.64E-03 |
| TENM2 | 50.98 | 10.94 | -2.22 | 4.33E-04 | 5.60E-03 |
| TFAP2B | 3.20 | 0.23 | -3.82 | 4.34E-03 | 3.38E-02 |
| THBS4 | 44508.49 | 15613.93 | -1.51 | 3.61E-04 | 4.81E-03 |
| THEMIS | 19.98 | 111.28 | 2.48 | 3.31E-08 | 2.27E-06 |
| THRB | 708.52 | 243.86 | -1.54 | 1.01E-14 | 8.20E-12 |
| THRSP | 57.77 | 7.26 | -2.99 | 3.77E-03 | 3.06E-02 |
| TIFAB | 3.96 | 33.98 | 3.10 | 4.83E-10 | 6.43E-08 |
| TIGIT | 44.51 | 318.72 | 2.84 | 7.43E-12 | 1.98E-09 |
| TLR10 | 9.29 | 125.53 | 3.76 | 4.81E-06 | 1.43E-04 |
| TMC2 | 3.11 | 0.23 | -3.78 | 5.12E-03 | 3.83E-02 |
| TMC5 | 7.57 | 2.16 | -1.81 | 5.95E-03 | 4.29E-02 |
| TMC8 | 293.54 | 996.19 | 1.76 | 7.28E-07 | 2.89E-05 |
| TMEM100 | 1580.38 | 458.74 | -1.78 | 4.08E-10 | 5.61E-08 |
| TMEM125 | 0.76 | 4.67 | 2.62 | 4.83E-03 | 3.67E-02 |
| TMEM132C | 160.63 | 9.92 | -4.02 | 3.77E-05 | 7.75E-04 |
| TMEM156 | 6.56 | 114.54 | 4.13 | 2.16E-07 | 1.07E-05 |
| TMEM163 | 21.16 | 76.09 | 1.85 | 2.85E-04 | 3.96E-03 |
| TMEM59L | 136.56 | 38.83 | -1.81 | 3.11E-05 | 6.64E-04 |
| TMPRSS3 | 0.45 | 8.02 | 4.16 | 3.48E-07 | 1.60E-05 |
| TNFRSF11B | 858.45 | 228.93 | -1.91 | 9.45E-06 | 2.51E-04 |
| TNFRSF13B | 1.33 | 29.24 | 4.46 | 3.99E-07 | 1.78E-05 |
| TNFRSF13C | 2.57 | 46.73 | 4.18 | 1.06E-03 | 1.13E-02 |
| TNFRSF17 | 3.46 | 165.87 | 5.59 | 3.56E-08 | 2.40E-06 |
| TNFRSF18 | 21.20 | 108.81 | 2.36 | 1.94E-07 | 9.86E-06 |
| TNFRSF4 | 29.51 | 157.31 | 2.41 | 3.55E-18 | 1.12E-14 |
| TNFRSF9 | 18.03 | 55.14 | 1.61 | 4.25E-05 | 8.58E-04 |
| TNFSF13B | 245.87 | 737.03 | 1.58 | 1.06E-10 | 1.83E-08 |
| TNFSF14 | 19.55 | 72.80 | 1.90 | 6.20E-10 | 7.85E-08 |
| TNNC1 | 17.06 | 4.79 | -1.83 | 3.37E-03 | 2.79E-02 |
| TNNT1 | 5.94 | 27.17 | 2.19 | 2.20E-03 | 2.02E-02 |
| TNXB | 54579.19 | 14213.29 | -1.94 | 3.12E-08 | 2.16E-06 |
| TOX | 40.69 | 126.54 | 1.64 | 1.03E-04 | 1.76E-03 |
| TPD52 | 90.97 | 520.79 | 2.52 | 1.84E-04 | 2.78E-03 |
| TRAF3IP3 | 99.13 | 459.24 | 2.21 | 4.27E-09 | 4.22E-07 |
| TRAT1 | 10.31 | 76.92 | 2.90 | 1.55E-07 | 8.18E-06 |
| TREML2 | 8.73 | 27.10 | 1.63 | 6.77E-03 | 4.73E-02 |
| TRHDE | 299.42 | 45.45 | -2.72 | 7.76E-10 | 9.46E-08 |
| TRIM55 | 0.40 | 8.96 | 4.50 | 6.21E-03 | 4.43E-02 |
| TRIM63 | 16.64 | 2.81 | -2.57 | 6.48E-03 | 4.59E-02 |
| TRPM3 | 38.78 | 12.86 | -1.59 | 1.01E-06 | 3.86E-05 |
| TSHR | 7.09 | 32.18 | 2.18 | 3.55E-03 | 2.92E-02 |
| TSTD1 | 20.57 | 61.86 | 1.59 | 4.93E-04 | 6.20E-03 |
| TTC16 | 8.62 | 25.47 | 1.56 | 8.28E-05 | 1.48E-03 |
| TTC24 | 5.53 | 28.75 | 2.38 | 1.62E-05 | 3.93E-04 |
| TUSC5 | 111.43 | 8.60 | -3.70 | 1.41E-04 | 2.26E-03 |
| UBASH3A | 12.99 | 118.02 | 3.18 | 1.95E-10 | 3.07E-08 |
| UBD | 30.59 | 634.77 | 4.38 | 1.60E-11 | 3.83E-09 |
| UNC13C | 3.70 | 0.39 | -3.24 | 3.02E-03 | 2.57E-02 |
| UNC5A | 5.28 | 22.71 | 2.10 | 5.39E-03 | 4.00E-02 |
| UNC5C | 455.87 | 136.78 | -1.74 | 6.11E-15 | 5.25E-12 |
| VIPR1 | 367.99 | 98.38 | -1.90 | 1.07E-07 | 5.99E-06 |
| VIPR2 | 56.43 | 15.82 | -1.83 | 2.73E-04 | 3.82E-03 |
| VPREB3 | 1.78 | 35.43 | 4.31 | 6.06E-04 | 7.29E-03 |
| VSIG1 | 3.02 | 14.73 | 2.28 | 4.35E-06 | 1.32E-04 |
| VSIG10L2 | 82.05 | 21.49 | -1.93 | 5.44E-05 | 1.05E-03 |
| VSTM2A | 10.09 | 0.10 | -6.62 | 2.41E-06 | 7.97E-05 |
| VWA2 | 24.75 | 6.55 | -1.92 | 3.00E-05 | 6.51E-04 |
| VWA3A | 12.67 | 3.35 | -1.92 | 1.46E-04 | 2.32E-03 |
| WDR17 | 81.64 | 23.31 | -1.81 | 8.82E-12 | 2.31E-09 |
| WFIKKN2 | 14.27 | 4.10 | -1.80 | 2.53E-04 | 3.60E-03 |
| WIF1 | 45.02 | 1.45 | -4.95 | 9.30E-06 | 2.48E-04 |
| WISP3 | 12.58 | 62.58 | 2.31 | 3.67E-03 | 3.00E-02 |
| WNK2 | 53.75 | 15.36 | -1.81 | 3.47E-07 | 1.59E-05 |
| WNT10A | 2.72 | 54.75 | 4.33 | 1.53E-11 | 3.70E-09 |
| WNT11 | 306.14 | 97.53 | -1.65 | 4.17E-10 | 5.67E-08 |
| WSCD2 | 302.60 | 81.33 | -1.90 | 9.11E-07 | 3.53E-05 |
| XBP1 | 2056.30 | 8382.56 | 2.03 | 2.04E-03 | 1.90E-02 |
| XCL1 | 5.83 | 28.07 | 2.27 | 1.99E-09 | 2.20E-07 |
| XCL2 | 6.97 | 38.95 | 2.48 | 5.80E-09 | 5.45E-07 |
| XCR1 | 26.98 | 106.87 | 1.99 | 5.83E-05 | 1.11E-03 |
| XG | 1050.38 | 355.73 | -1.56 | 2.35E-13 | 1.30E-10 |
| XKR4 | 9.86 | 2.42 | -2.03 | 1.01E-03 | 1.09E-02 |
| ZAP70 | 90.97 | 570.15 | 2.65 | 3.23E-08 | 2.22E-06 |
| ZBED2 | 0.92 | 25.29 | 4.78 | 1.24E-08 | 1.02E-06 |
| ZBED6CL | 21.47 | 66.90 | 1.64 | 3.66E-06 | 1.14E-04 |
| ZBP1 | 21.54 | 344.08 | 4.00 | 3.83E-07 | 1.72E-05 |
| ZBTB32 | 1.68 | 29.50 | 4.13 | 1.03E-08 | 8.76E-07 |
| ZIC1 | 650.88 | 224.30 | -1.54 | 9.21E-07 | 3.55E-05 |
| ZNF365 | 233.78 | 76.41 | -1.61 | 4.10E-08 | 2.70E-06 |
| ZNF488 | 32.10 | 9.35 | -1.78 | 3.73E-06 | 1.16E-04 |
| ZNF534 | 10.94 | 2.71 | -2.01 | 7.78E-04 | 8.92E-03 |
| ZNF536 | 9.64 | 2.59 | -1.90 | 1.59E-03 | 1.56E-02 |
| ZNF683 | 4.41 | 19.92 | 2.18 | 1.67E-05 | 4.01E-04 |
| ZNF728 | 12.64 | 4.13 | -1.61 | 2.20E-03 | 2.03E-02 |
| ZNF80 | 1.28 | 7.95 | 2.63 | 1.86E-03 | 1.76E-02 |
| ZNF831 | 26.16 | 160.63 | 2.62 | 7.51E-09 | 6.74E-07 |
| ZNF98 | 8.51 | 2.16 | -1.98 | 1.18E-03 | 1.23E-02 |

**Supplementary Table3 The GO Functional Enrichment Analysis**

| Category | Term | Count | FDR |
| --- | --- | --- | --- |
| MF | heparin binding | 21 | 1.92×10^-3^ |
| MF | cytokine activity | 22 | 2.01×10^-03^ |
| MF | receptor activity | 29 | 2.37×10^-05^ |
| MF | receptor binding | 40 | 1.26×10^-05^ |
| MF | calcium ion binding | 63 | 1.26×10^-05^ |
| BP | cell-cell signaling | 35 | 2.11×10^-06^ |
| BP | inflammatory response | 52 | 5.76×10^-10^ |
| BP | cell adhesion | 60 | 8.59×10^-11^ |
| BP | immune response | 79 | 1.65×10^-24^ |
| BP | signal transduction | 94 | 3.01×10^-06^ |
| CC | extracellular space | 108 | 2.33×10^-08^ |
| CC | extracellular region | 131 | 7.63×10^-11^ |
| CC | integral component of plasma membrane | 156 | 3.32×10^-26^ |
| CC | integral component of membrane | 322 | 7.39×10^-13^ |
| CC | plasma membrane | 327 | 1.91×10^-31^ |

**Supplementary Table4** **The KEGG pathway analysis**

| Category | Term | Count | FDR |
| --- | --- | --- | --- |
| KEGG | Cytokine-cytokine receptor interaction | 52 | 3.05×10^-17^ |
|  | Cell adhesion molecules (CAMs) | 31 | 6.87×10^-10^ |
|  | Chemokine signaling pathway | 31 | 3.50×10^-07^ |
|  | Neuroactive ligand-receptor interaction | 31 | 6.08×10^-04^ |
|  | Hematopoietic cell lineage | 18 | 3.10×10^-05^ |
|  | T cell receptor signaling pathway | 17 | 5.17×10^-04^ |
|  | Rheumatoid arthritis | 16 | 5.17×10^-04^ |
|  | Intestinal immune network for IgA production | 15 | 1.30×10^-06^ |
|  | Primary immunodeficiency | 14 | 2.13×10^-07^ |
|  | Autoimmune thyroid disease | 12 | 6.08×10^-04^ |
|  | Viral myocarditis | 12 | 1.30×10^-03^ |
|  | Allograft rejection | 11 | 2.40×10^-04^ |
|  | Type I diabetes mellitus | 11 | 5.17×10^-04^ |
|  | Inflammatory bowel disease (IBD) | 11 | 1.48×10^-02^ |
|  | Antigen processing and presentation | 11 | 4.58×10^-02^ |
